# Potent In Vitro and In Vivo Anticancer Activity of New Bipyridine and Bipyrimidine Gold (III) Dithiocarbamate Derivatives

**DOI:** 10.3390/cancers11040474

**Published:** 2019-04-04

**Authors:** Muhammad Altaf, Naike Casagrande, Elena Mariotto, Nadeem Baig, Abdel-Nasser Kawde, Giuseppe Corona, Roberto Larcher, Cinzia Borghese, Claudia Pavan, Adam A. Seliman, Donatella Aldinucci, Anvarhusein A. Isab

**Affiliations:** 1 Department of Chemistry, GC University, Lahore 54000, Pakistan; muhammad.altaf@gmail.com; 2 Molecular Oncology, Centro di Riferimento Oncologico di Aviano (CRO) IRCCS, 33081 Aviano, Italy; naike.casagrande@libero.it (N.C.); mariottoelena@gmail.com (E.M.); cpborghese@cro.it (C.B.); c.pavan@outlook.com (C.P.); 3 Department of Chemistry, King Fahd University of Petroleum and Minerals, Dhahran 31261, Saudi Arabia; nadeembaig@kfupm.edu.sa (N.B.); akawde@kfupm.edu.sa (A.-N.K.); 4 Immunopathology and Cancer Biomarkers, Centro di Riferimento Oncologico di Aviano (CRO) IRCCS, 33081 Aviano, Italy; giuseppe.corona@cro.it; 5 Center for Technological Transfer, Edmund Mach Foundation, 38010 Trento, Italy; roberto.larcher@fmach.it; 6 Lab Technical Support Office (LTSO), King Fahd University of Petroleum and Minerals, Dhahran 31261, Saudi Arabia; adamchemi@gmail.com

**Keywords:** gold (III) complexes, anticancer therapy, cisplatin resistance, doxorubicin resistance, prostate cancer

## Abstract

We synthesized eight new bipyridine and bipyrimidine gold (III) dithiocarbamate-containing complexes (**C1**–**C8)** and tested them in a panel of human cancer cell lines. We used osteosarcoma (MG-63), lung (A549), prostate (PC3 and DU145), breast (MCF-7), ovarian (A2780 and A2780cis, cisplatin- and doxorubicin-resistant), and cervical (ME-180 and R-ME-180, cisplatin resistant) cancer cell lines. We found that **C2**, **C3**, **C6**, and **C7** were more cytotoxic than cisplatin in all cell lines tested and overcame cisplatin and doxorubicin resistance in A2780cis and R-ME-180 cells. In the PC3 prostate cancer cell line, the gold (III) complex **C6** ([Au_2_(BPM)(DMDTC)_2_]Cl_4_) induced apoptosis and double-stranded DNA breaks, modified cell cycle phases, increased Reactive Oxigen Species (ROS) generation, and reduced thioredoxin reductase and proteasome activities. It inhibited PC3 cell migration and was more cytotoxic against PC3 cells than normal human adipose-derived stromal cells. In mice bearing PC3 tumor xenografts, **C6** reduced tumor growth by more than 70% without causing weight loss. Altogether, our results demonstrate the anticancer activity of these new gold (III) complexes and support the potential of **C6** as a new agent for prostate cancer treatment.

## 1. Introduction

Cisplatin and a few related platinum compounds, such as carboplatin and oxaliplatin, are common anticancer agents [1,2], but their use often causes significant toxicity and leads to drug resistance [3,4]. Therefore, other metallodrugs [5] containing platinum or non-platinum metals, such as ruthenium, palladium, titanium, gold, and copper, have been investigated [6,7,8]. In particular, gold (I) and gold (III) complexes have been widely studied and found to have anticancer effects in vitro and in vivo [5,8,9].

Gold (I) and gold (III) complexes have a variety of mechanisms of action [9], including inhibition of the enzyme thioredoxin reductase (TrxR), increased generation of reactive oxygen species (ROS) [10,11], proteasome inhibition [12], interaction with DNA [13], alteration of the cell cycle phases [14], and modulation of kinases [15]. These multifaceted modes of action enable gold complexes to exert potent cytotoxicity against cancer cells, including multidrug-resistant tumor cells [5,9]. However, gold and other heavy metals such as platinum also react with the sulfur-containing amino acids cysteine (a thiol) and methionine (a thioether), generating metal‒protein adducts that can be nephrotoxic [9]. 

One way to prevent interactions between the metal center of anticancer drugs and thiol-containing biomolecules is to use dithiocarbamate as a chelating ligand. Through its sulfur atoms, dithiocarbamate coordinates metal ions, thereby stabilizing metal drugs and reducing interactions with biomolecules [5,16]. Some of us reported that gold (III) dithiocarbamate complexes had potent in vitro anticancer activity against acute myeloid leukemia cells [11] and prostate cancer cells [17] and low toxicity in tumor-bearing mice [17]. To improve the delivery and cellular uptake of these compounds, we designed a second generation of gold (III) dithiocarbamate complexes [18]. These molecules, called peptidomimetics, are derivatives of oligopeptides, and they were designed to target the peptide transporters PEPT1 and PEPT2 that are upregulated in several tumor types. The compounds showed promising anticancer activity in different tumor models [18], including breast [19] and prostate cancer [20]. 

In parallel research, a team led by Altaf designed and synthesized other metallodrugs with anticancer activity, including gold (I) [21,22] and gold (III) complexes [23]. Together, our two research groups produced new bipyridine gold (III) dithiocarbamate complexes with nitrogen and sulfur donor ligands [24]. The new gold (III) complexes were cytotoxic in cisplatin-resistant ovarian carcinoma cells as well as in p53-defective cancer cells of different tumor types, and compound **1** was shown to be less cytotoxic in non-cancer human mesenchymal stromal cells than in cancer cells [24]. To improve upon the antitumor properties of gold (III) analogs, we synthesized eight new gold (III) dithiocarbamate complexes with bipyridine or bipyrimidine ligands. Here, we report the synthesis, chemical analysis, and anticancer activity of these new gold (III) complexes (**C1**–**C8**). We screened the compounds on a panel of human cancer cell lines, including doxorubicin- and cisplatin-resistant cells, and evaluated the mechanism of action and the in vivo anticancer activity of the most active compound **C6** ([Au_2_(BPM)(DMDTC)_2_]Cl_4_) against PC3 prostate cancer cells. 

## 2. Results

**Figure 1:**
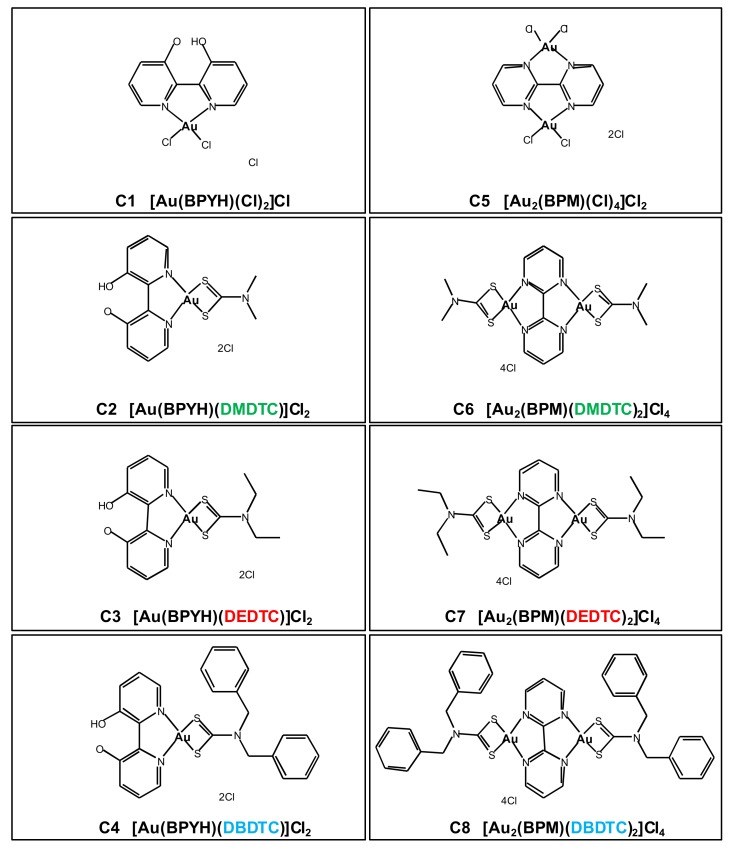
Chemical structures of the eight new gold (III) complexes (compounds **C1**–**C8**). BPYH, 2,2′-bipyridine-3,3′-diol; BPM, 2,2′-bipyrimidine; DMDTC, dimethyldithiocarbamate; DEDTC, diethyldithiocarbamate; DBDTC, dibenzyldithiocarbamate.

The expected structures of eight new gold (III) compounds (**C1**–**C8**) are shown in Figure 1. Four molecules (compounds **C1–C4)** have a 2,2′-bipyridine-3,3′-diol (BPYH) moiety and a single gold atom, while the others (compounds **C5‒C8**) have a 2,2′-bipyrimidine (BPM) moiety and two gold atoms. Compounds **C2** and **C6** are dimethyldithiocarbamates, **C3** and **C7** are diethyldithiocarbamates, and **C4** and **C8** are dibenzyldithiocarbamates. The gold (III) complexes had >99% purity and interacted with lysozyme, tryptophan, and guanine (Supplementary Materials, Figures S1–S10 and Tables S1–S3).

### 2.1. In Vitro Cytotoxicity of Gold (III) Complexes

To evaluate the potential anticancer activity of the eight compounds, we first compared their in vitro cytotoxicity to that of cisplatin in a panel of cell lines derived from different human cancers including lung cancer (A549), androgen-sensitive prostate cancer (DU145), androgen-resistant prostate cancer (PC3), breast cancer (MCF-7), and osteosarcoma (MG-63) (Table 1).

Cisplatin had relatively low potency on three cell lines (A549, MCF-7, and MG-63), with half maximal inhibitory concentrations (IC_50_) >10 µM, while it was more potent on the DU145 and PC3 prostate cancer cell lines with mean IC_50_ values of 4.5 µM and 3.3 µM, respectively. Compound **C1** had IC_50_ values higher than that of cisplatin in most cell lines, indicating lower potency, while in MG-63 the two drugs had similar activities. Compounds **C2** and **C3** were more potent than cisplatin in all cell lines and had IC_50_ values more than one order of magnitude lower than cisplatin in the cisplatin-resistant MCF-7, A549, and MG-63 cell lines. Compounds **C4** and **C8** were less cytotoxic than cisplatin in PC3 and DU145 cells but more cytotoxic than cisplatin in MCF-7, A549, and MG-63 cells. Compound **C5** exerted very low cytotoxic effects on most cell lines except for MG-63. Compound **C6** and **C7** had submicromolar IC_50_ values in almost all cell lines, and thus were the most potent of all compounds tested, including cisplatin.

The cytotoxic effects of the gold (III) compounds were also evaluated in two cell lines for which a cisplatin-resistant clone was available. First, in the ovarian cancer cell line A2780 (cisplatin sensitive), compounds **C2**, **C3**, **C6**, and **C7** were more potent than cisplatin (i.e., they had IC_50_ values <1.5 µM), whereas compounds **C1**, **C4**, **C5**, and **C8** were less potent (Table 2).

In the cisplatin- and doxorubicin-resistant clone A2780cis, the IC_50_ of each gold (III) compound was similar to that in the parental cell line, but because the IC_50_ of cisplatin was higher, compounds **C2**, **C3**, **C4**, **C6**, **C7**, and **C8** were all more potent than the reference drug. The fold resistance (FR) between the two cell lines (IC_50_ A2780cis/IC_50_ A2780) was 6.9 for cisplatin and 9.0 for doxorubicin, while for the eight test compounds it was close to unity (range, 0.9 to 1.4). This result excludes the phenomenon of cross-resistance to these two drugs in these cell lines.

A similar experiment was carried out using the ME-180 cervical cancer cell line and its cisplatin-resistant clone R-ME-180 (Table 3). In ME-180 cells, compounds **C2**, **C3**, **C6**, and **C7** were more potent than cisplatin (i.e., they had IC_50_ values <15 µM), and compounds **C1**, **C4**, and **C5** were less active. In R-ME-180 cells, the IC_50_ value for cisplatin was higher, giving FR = 4.5. The FR for the test compounds (excluding **C5**) was lower, ranging from 0.9 (**C7**) to 1.5 for **C2**.

A similar experiment was carried out using the ME-180 cervical cancer cell line and its cisplatin-resistant clone R-ME-180 (Table 3). In ME-180 cells, compounds **C2**, **C3**, **C6**, and **C7** were more potent than cisplatin (i.e., they had IC_50_ values <15 µM), and compounds **C1**, **C4**, and **C5** were less active. In R-ME-180 cells, the IC_50_ value for cisplatin was higher, giving FR = 4.5. The FR for the test compounds (excluding **C5**) was lower, ranging from 0.9 (**C7**) to 1.5 for **C2**.

Then, we examined the cellular uptake of the gold (III) compounds. PC3 cells were incubated separately with two low-potency compounds **C4** and **C5** and two high-potency compounds **C6** and **C7**, and the amount of internalized gold was determined using mass spectrometry (Figure S11). This analysis showed greater uptake of the two more potent molecules, with **C6** internalization even greater than that of **C7**. 

To further investigate the cytotoxicity of **C6** ([Au_2_(BPM)(DMDTC)_2_]Cl_4_ ), we compared its effects on growth of PC3 cells and normal human adipose-derived stromal cells. This analysis showed that **C6** was more potent in the prostate tumor cells (IC_50_ = 0.6 μM) than in the normal stromal cells (IC_50_ = 1.4 μM) (Figure S12).

Altogether, these experiments show that compounds **C2**, **C3**, **C6**, and **C7** have the greatest potency (lowest IC_50_ values) in the panel of investigated tumor cell lines. Results from pairs of cell lines that differ in susceptibility to cisplatin (ME-180 and R-ME180) and also to doxorubicin (A2780 and A2780cis) rule out cross-resistance to the two chemotherapy agents.

### 2.2. Cellular Mechanism of Action of Compound ***C6***

**Figure 2:**
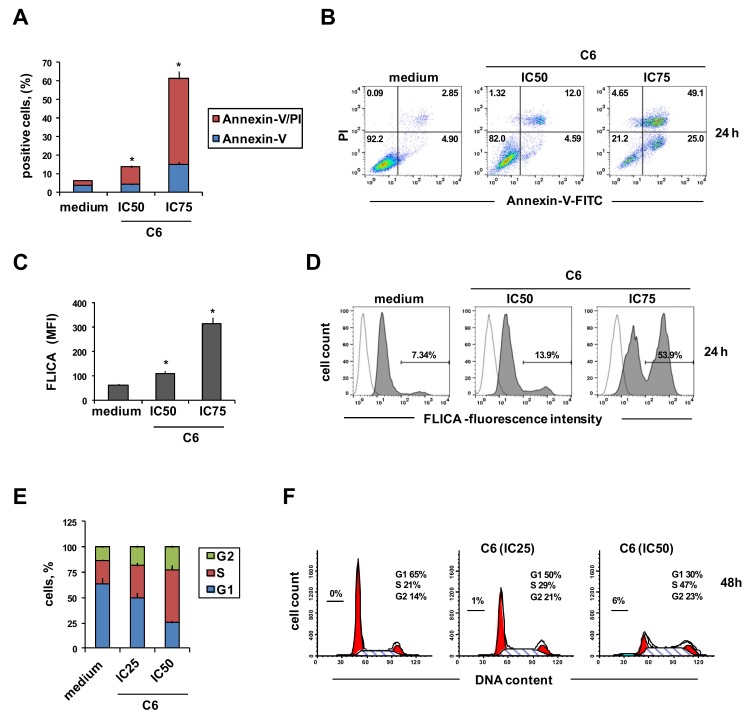
**C6** induces apoptosis and blocks the cell cycle. PC3 cells were cultured in complete medium alone or with **C6**, at the half-maximal inhibitory concentration (IC_50_), at IC_25_, or IC_75_, for 24 h. (**A**,**B**) Annexin-V-FITC and propidium iodide (PI) assay of apoptosis by flow cytometry. (**A**) Percentage of annexin-V-positive cells. (**B**) Representative flow cytometry plots; the percentages of stained cells are reported. (**C**,**D**) Caspase 3,7 activation assay evaluated using flow cytometry with fluorochrome-labeled inhibitors of caspases (FLICA). (**C**) Mean fluorescence intensity (MFI) of FLICA. (**D**) Representative flow cytometry histograms of caspase 3,7 activation by **C6** treatment. The percentage of FLICA-positive cells is reported. (**E**,**F**) Cell cycle progression determined by PI staining. (**E**) Percentage distribution of cells in different cell cycle phases. (**F**) Representative flow cytometry histograms of cell cycle progression. All bar charts report means and SD of three independent experiments. Statistical analysis was performed using one-way ANOVA, followed by Dunnett’s multiple comparisons test. * *p* < 0.05 vs. medium.

**C6** was chosen for further analyses with the PC3 prostate cancer cell line. When PC3 cells were incubated with **C6** at IC_50_ (0.62 µM) and IC_75_ (1.85 µM), there was a dose-dependent increase in the percentage of annexin-V-positive cells, indicating early apoptosis, and also of double stained annexin-V- and propidium iodide (PI)-positive cells, indicating late apoptosis (Figure 2A,B). Consistently, treatment with **C6** activated caspase 3,7, evaluated using fluorochrome-labeled inhibitors of caspases (FLICA) that irreversibly bind active caspase (Figure 2C,D). These results suggest that apoptosis is involved in tumor cell death by **C6**. Finally, treatment of PC3 cells with **C6** modified the distribution of cells in the cell cycle, by increasing the percentage of cells in S phase and decreasing that in G1 compared to untreated cells (Figure 2E,F).

**Figure 3:**
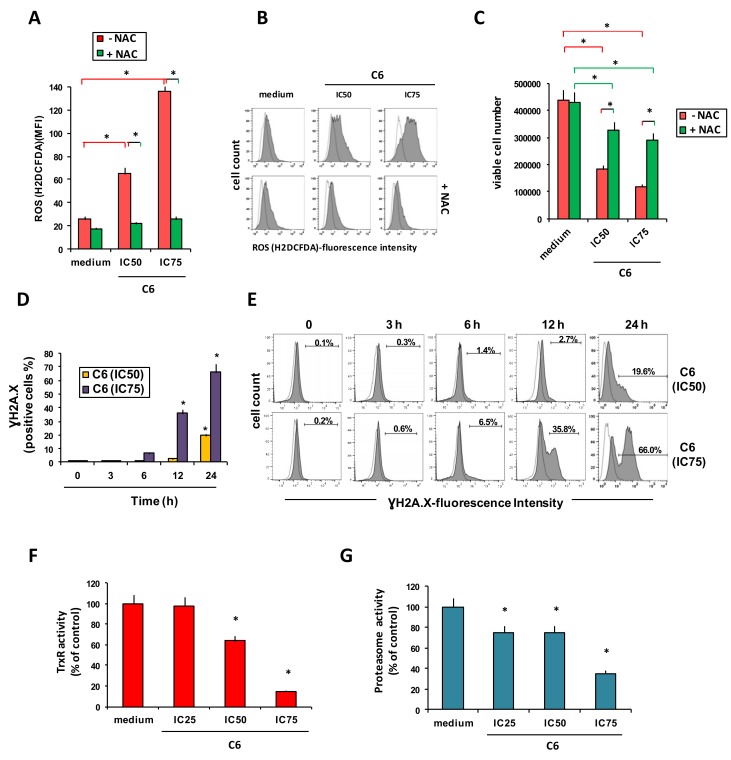
Compound **C6** increases ROS generation induces double-stranded DNA breaks and reduces thioredoxin reductase and 20 S proteasome activities. PC3 cells were cultured in complete medium alone or with compound **C6**, at the half-maximal inhibitory concentration (IC_50_), at IC_25_, or IC_75_. (**A**,**B**) Reactive oxygen species (ROS) generation by PC3 cells exposed to **C6** at IC_50_ and IC_75_ for 24 h, in the presence or absence of the ROS scavenger N-acetyl cysteine (NAC) (added 30 min before drug treatment). ROS were detected using H2DCFDA. (**A**) Generated ROS levels (mean fluorescence intensity, MFI). (**B**) Representative flow cytometry histograms of generated ROS. (**C**) Number of viable cells 24 h after treatment with **C6** and NAC (NAC, added 30 minutes before drug treatment), evaluated using trypan blue dye exclusion. (**D**,**E**) Histone H2A.X phosphorylation (γH2A.X) as a measure of double-stranded DNA breaks, detected with FITC anti-H2A.X Phospho (Ser139) antibody. (**D**) Time course of phosphorylation at two concentrations of **C6**. (**E**) Representative flow cytometry histograms of γH2A.X. (**F**) Thioredoxin reductase (TrxR) activity after a 12 h treatment with **C6**, normalized to control (medium). (**G**) Proteasome activity after a 12 h treatment with **C6**, evaluated with the 20S-Proteasome Assay kit, normalized to control (medium). All bar charts report means and SD of three independent experiments. Statistical analysis was performed using one-way ANOVA, followed by Turkey’s or Dunnett’s multiple comparisons test where appropriate. * *p* < 0.05 vs. medium, unless otherwise indicated.

Next, we determined if **C6** treatment led PC3 cells to increase the production of reactive oxygen species (ROS). Two concentrations of **C6** induced ROS production in a dose-dependent manner, and this effect was blocked when cells were pretreated with *N*-acetyl cysteine (NAC), a ROS scavenger (Figure 3A,B).

NAC decreased the cytotoxic effects of **C6** (Figure 3C), suggesting that ROS generation is involved in this compound’s cytotoxicity. Treatment of PC3 cells with **C6** also induced, in a time- and dose-dependent manner, double-stranded DNA breaks, as shown by an increase in phosphorylation of histone H2A.X (Figure 3D,E). We did not observe H2AX phosphorylation at the early time points (Figure 3D,E). Because ROS elimination and the maintenance of intracellular redox balance depend on the thioredoxin (Trx) system [25], we examined the effects of **C6** used at IC_25_ (0.31 µM), IC_50_ (0.62 µM), and IC_75_ (1.85 µM) on Trx reductase (TrxR) levels and found that a short incubation resulted in a dose-dependent decrease of its enzymatic activity (Figure 3F). Finally, **C6** exerted a dose-dependent inhibitory effect also on 20S proteasome activity (Figure 3G).

### 2.3. Effects of ***C6*** on Tumor Cell Migration and Xenograft Growth

**Figure 4:**
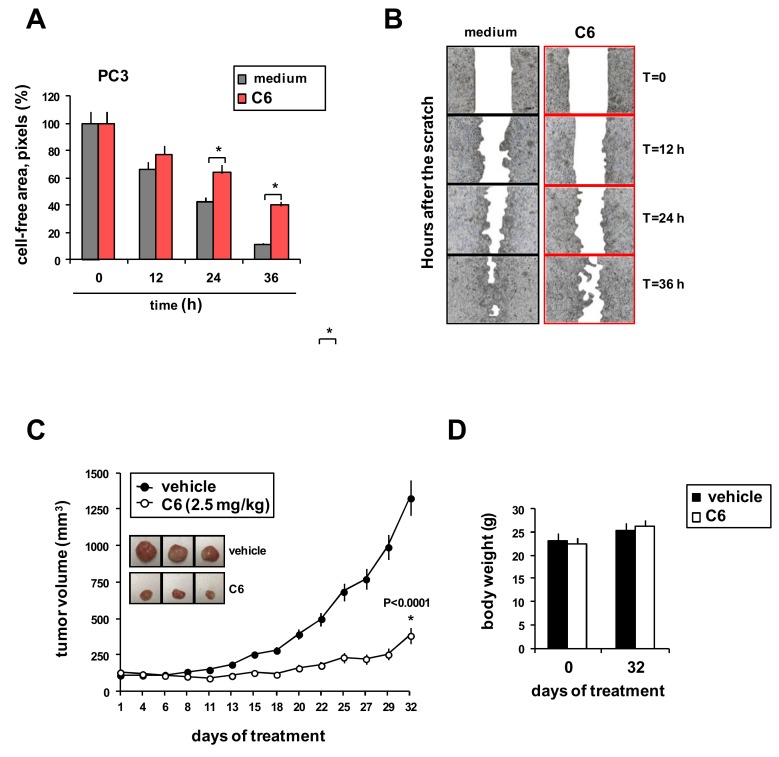
Compound **C6** reduces cell migration and PC3 tumor xenograft growth. (**A**,**B**) Scratch assay. Confluent monolayers of PC3 cells were treated or not with compound **C6** (IC_50_) for 3 h in complete medium, “wounded” by scraping, then cultured in low serum medium and photographed every 12 h for up to 36 h. (**A**) Cell-free area remaining over time as cells migrated into the wound, normalized to time 0. Bar graph shows the mean and SD of three experiments each done in triplicate. * *p* < 0.05, Student’s *t* test. (**B**) Representative phase contrast photomicrographs, original magnification 4×. (**C**) Growth of xenografts in nude mice inoculated with PC3 cells (3 × 10^6^ cells/animal) and treated intratumorally with **C6** (2.5 mg/kg) (*n* = 5) or vehicle (*n* = 5). Values are mean and SD. Student’s *t* test, * *p* < 0.0001. (**D**) Body weights of xenografted mice (*n* = 5 per group).

The effect of **C6** on PC3 cell migration was evaluated using the in vitro scratch assay. A 3 h pretreatment slowed the ability of PC3 cells to refill an empty area (“scratch”) of the monolayer compared to untreated cells: 36 h after the monolayer was scratched, the remaining uncovered area was about 40% in **C6**-pretreated cells and about 10% in control cells (Figure 4A,B). Finally, we examined the effects of **C6** on the in vivo growth of PC3 cell xenografts in female athymic nude mice. Inhibition of tumor growth became apparent starting 15 days after the beginning of treatment, compared to animals not treated with **C6** (Figure 4C). By day 32, control tumors had grown to a mean volume of 1327 mm^3^ (SD = 105 mm^3^) whereas **C6**-treated tumors reached 385 mm^3^ (SD = 35 mm^3^), reflecting a 71% inhibitory effect (Figure 4C). This difference was significant (*p* < 0.0001, Student’s *t* test). **C6** treatment did not affect the weight of the animals (Figure 4D).

## 3. Discussion

In this study, we evaluated the anticancer activity of new bipyridine and bipyrimidine gold (III) complexes (**C1**–**C8**) using a panel of cancer cell lines. The eight new compounds had potent cytotoxicity in ovarian, lung, breast, prostate, cervical and sarcoma cancer cell lines. They were also active in a cisplatin-resistant cervical cell line (R-ME-180) and in a cisplatin- and doxorubicin-resistant ovarian cancer cell line (A2780cis), suggesting that they may overcome both cisplatin and doxorubicin resistance.

The mechanism of action and the in vivo activity of the most active compound, **C6**, were evaluated using androgen-resistant PC3 prostate cancer cells. **C6** induced apoptosis, activated caspases 3,7, and modified the distribution of cells in cell cycle phases. Moreover, **C6** increased ROS generation. ROS may play an important role in the cytotoxic effect of **C6** since the ROS scavenger NAC counteracted **C6**’s ability to inhibit cell growth. **C6** treatment also induced double-stranded DNA breaks. This DNA damage is more likely due to apoptosis induced by the increased intracellular ROS levels [25] than to a direct effect of **C6** on DNA. Thioredoxin (Trx) and the seleno-enzyme thioredoxin reductase (TrxR) are essential components of the Trx system that regulates cellular redox signaling pathways [25,26]. TrxR inhibition increases ROS accumulation, which causes mitochondrial dysfunction and apoptosis [25]. High levels of Trx and TrxR have been found in many different tumor types, including prostate cancer [27], and are associated with tumor progression and resistance to several anticancer drugs, including cisplatin [28]. For these reasons, the Trx system may be a target for cancer therapy [25,26]. TrxR has already been identified as an important target of several gold (I) (e.g., auranofin) [10,29] and gold (III) complexes [15,17,20]. Here we found that, consistently with increased ROS generation, **C6** inhibited TrxR enzymatic activity in PC3 cells.

The proteasome, a central component of the protein degradation machinery, controls the expression of proteins linked to cell survival and proliferation [30]. Cancer cells produce anti-apoptotic and pro-survival proteins, and their treatment with proteasome inhibitors causes cell cycle arrest or apoptosis, suggesting their use in clinic [31]. Some gold (III) complexes have already been found to target the proteasome in cancer cells [8,12,17,20,32,33], and here we found that **C6** inhibited proteasome activity in prostate cancer cells.

Since androgen-independent prostate cancer has high invasive potential, a successful therapeutic approach should counteract not only tumor growth but also the metastatic potential [34]. Here, we found that **C6** reduced PC3 cell migration, suggesting that this gold (III) complex may inhibit not only tumor proliferation, but also its dissemination. The adverse effects of **C6** on cell migration may be attributed to a decreased expression of molecules involved in PC3 cell migration or to decreased growth of treated cells.

Some studies of metal-based compounds, including gold (III) complexes, have found promising in vitro cytotoxicity but did not test growth inhibition in in vivo experiments [8,35,36]. Therefore, we evaluated the in vivo antitumor activity of **C6**. Consistent with our in vitro studies, **C6** significantly reduced PC3 tumor xenograft growth with low toxicity (measured as body weight change). 

## 4. Materials and Methods

Methods for the synthesis and chemical characterization of the gold (III) complexes are described in the Supplementary Materials, together with electrochemical methods for testing their interactions with a protein, an amino acid, and a nucleobase and cellular methods for testing uptake 

### 4.1. Drugs

Gold (III) complexes were dissolved in DMSO to 10 µM. The same amount of DMSO necessary to dissolve the compounds was used as negative control in all experiments. Cisplatin and doxorubicin were surplus drugs obtained from the pharmacy at Centro Riferimento Oncologico. 

### 4.2. Cell Lines and Culture Conditions

Human androgen-resistant (PC3) and androgen-sensitive (DU145) prostate cancer cell lines were obtained from the German Collection of Microorganisms and Cell Cultures (DSMZ, Braunschweig, Germany). Human breast adenocarcinoma MCF-7 (HTB-22TM), lung cancer (A549), and osteosarcoma (MG-63) cell lines were from the American Type Culture Collection (ATCC, Rockville, MD, USA). Human ovarian epithelial carcinoma-derived A2780 cell line and its cisplatin- and doxorubicin-resistant clone A2780cis were from Sigma-Aldrich (Milano, Italy). The highly invasive cervical cancer-derived ME-180 (HPV positive) cell line was a kind gift of Dr. G. Toffoli (CRO, Aviano, Italy), and the cisplatin-resistant clone R-ME-180 was developed in our laboratory by continuous exposure to 1 µM cisplatin. Cell lines were tested for mycoplasma every 15 d using the MycoAlert test (Lonza, Verviers, Belgium).

A549, MG-63, MCF-7, ME-180, and R-ME-180 cells were cultured in DMEM, and A2780, A2780cis, PC3, and DU145 cells were cultured in RPMI-1640 medium; media contained 10% heat-inactivated fetal bovine serum (FBS), 1% (v/v) of penicillin (10,000 units/mL)-streptomycin (10 mg/mL), and 1% (v/v) L-glutamine (200 mM) (all from Sigma-Aldrich). R-ME-180 and A2780cis cells were maintained in 1 μM cisplatin. Adipose-derived stromal cells were maintained in Mesenchymal-Stem-Cell Growth Medium Bulletkit MSCGM (Lonza). All cell lines were cultured at 37 °C in a 5% CO_2_, fully humidified atmosphere.

### 4.3. Cytotoxicity Assay

Cell lines were seeded in 96-well flat-bottomed microplates in 100 μL culture medium at the following densities: DU145, PC3, and MCF-7 cells (2.5 × 10^3^ cells/well); A2780, A2780cis, ME-180, R-ME-180, and A549 cells (4.0 × 10^3^ cells/well); and MG-63 cells (2.0 × 10^3^ cells/well). Cells were allowed to adhere for 24 h. Then, the medium was replaced with fresh medium alone or with one of the gold (III) compounds at increasing concentrations from 0 to 100 μM. The reference drugs cisplatin (0–100 μM) and doxorubicin (0–1 μM) were included as positive controls for growth inhibition. After 72 h, cell viability was assayed using the 3-(4,5-dimethylthiazol-2-yl)-2,5-diphenyltetrazolium bromide (MTT) assay. All experimental conditions were tested in triplicate and the experiment was done three times. 

Half maximal inhibitory concentrations (IC_50_, the concentration required for 50% in vitro inhibition of growth) and IC_25_ and IC_75_ values were calculated for each experiment using CalcuSyn software (Version 2, Biosoft, Ferguson, MO, USA) [37]. IC_50_ values were reported as mean (SD). For drug-resistance cell lines, fold resistance (FR) was calculated as the ratio of the IC_50_ of the resistant cell line to the IC_50_ of the parental cell line.

### 4.4. Cellular Assays

In all cellular assays, PC3 cells (2.0 × 10^5^ cells/well in six-well plates) were incubated in complete culture medium containing different concentrations of compound **C6** (IC_25_ = 0.31 µM, IC_50_ = 0.62 µM, IC_75_ = 1.85 µM). All experimental conditions were tested in triplicate and experiments were done three times to calculate means and SD. 

For apoptosis assays, PC3 cells were treated with **C6** for 24 h and then apoptosis was assayed by staining for 15 min with FITC Annexin V reagent (BD Bioscences, Milano, Italy) and propidium iodide (PI). Apoptotic cells were detected using flow cytometry (BD FACSCanto II flow cytometer) and analyzed using BD FACSDiva v8.0.1 software (BD Biosciences, Milano, Italy). Caspase 3,7 activation was evaluated using fluorochrome-labeled inhibitors of caspases (FLICA) of the CaspaTag Caspase 3,7 In Situ Assay Kit, Fluorescein (Chemicon-Millipore International, Milan, Italy) and evaluated using flow cytometry; data were expressed as mean fluorescence intensity.

To assay the distribution of cells in the various phases of the cell cycle, PC3 cells were treated with **C6** for 48 h, then harvested, fixed in cold 70% ethanol for 15 min, and stained with PI solution (50 µg/mL PI, 0.1% NP-40, 100 µg/mL PureLink RNase A, 0.1% sodium citrate (Sigma-aldrich, Milano, Italy). After 1 h, cells were analyzed using flow cytometry. The distribution of cells in different cell cycle phases was quantified using ModFit LT 4.0 software (BD Biosciences, Milano, Italy). The production of reactive oxygen species (ROS) was evaluated using 2′,7′-dichlorodihydrofluorescein diacetate (H2DCFDA) (H2-DCF, DCF) (Invitrogen, Monza, Italy). Cells were pretreated with the antioxidant N-acetyl-L-cysteine (NAC; 5 mM) (Sigma, Milano, Italy) for 30 min before **C6** was added. After 24 h of **C6** treatment, cells were harvested, and viable cells were counted using trypan blue dye exclusion. Then, cells were washed, stained with 1 µM H2DCFDA for 30 min at 37 °C, and finally ROS production was analyzed using flow cytometry. 

The presence of double-stranded DNA breaks was assessed 3, 6, 12, and 24 h after treatment with **C6** by fixing and permeabilizing cells with Fix & Perm medium A and B (Invitrogen) and staining with FITC anti-H2A.X Phospho (Ser139) Antibody (BioLegend, San Diego, CA, USA). Stained cells were evaluated using flow cytometry.

Thioredoxin reductase (TrxR) (EC 1.8.1.9) was assayed using the Thioredoxin Reductase Assay Kit (Sigma-Aldrich). Cells were treated with **C6** for 12 h and then lysed in 50 mM Tris-HCl pH 7.6, 0.1% Triton X-100, 0.9% NaCl. Enzyme activity was determined reading absorbance at 412 nm using a spectrophotometer (Biomate 3 Thermo Spectronic, Thermo Electonic Corporation, Monza, Italy). The enzymatic activity was normalized to the protein concentration, determined using the Bio-Rad protein assay (Protein Assay Dye Reagent Concentrate, Bio-Rad Laboratories, Segrate, Italy), and expressed as percentage of control (no **C6**).

Proteasome activity (EC 3.4.25.1) was evaluated on the same cell lysates as used to assay TrxR. Proteasome activity was assayed in cytosolic extracts using the 20S Proteasome Activity Assay kit APT280 (Merck Millipore) and a computer-interfaced GeniusPlus microplate reader (Tecan Trading AG, Switzerland). The activity was normalized to the protein concentration, determined using the Bio-Rad protein assay, as expressed as percentage of control. 

Cell migration was assessed using the in vitro scratch assay. Briefly, cells were grown to confluence and then treated with **C6** (IC_50_). After 3 h, monolayers were washed twice with PBS, scraped with a pipette tip to create a “wound” in the monolayer, and washed again. Culture medium with 2% (not 10%) FBS was added and the cells were cultured for 36 h. Wounds were photographed every 12 h using an inverted microscope (EclipseTS/100, Nikon, Instruments Europe BV Amsterdam, Netherlands) at magnification 4×. Migration was assessed by measuring the cell-free area (in pixels) with ImageJ-NIH (National Institutes of Health) tool software after 12, 24, and 36 h. 

### 4.5. Human Prostate Tumor Xenograft Experiments

Animal experiments were approved by the Italian Ministry of Health (no. 671/2015/PR). Ten 4-week-old female athymic nu/nu (nude) mice were purchased from Envigo (Udine, Italy). PC3 cells (3 × 10^6^ in a 0.1 mL solution of Matrigel 1:3 in PBS) were inoculated subcutaneously into the right flank of each mouse. Body weight and tumors were measured three times a week, and tumor volumes were calculated according to the formula: (width^2^ × length × 3.14)/6. When tumors reached a volume of ca. 120 mm^3^, mice were divided into two groups of five animals each. Mice were treated every other day with an intratumoral injection of 2.5 mg/kg **C6** or an equal volume of vehicle (10% DMSO, 20% Cremophor Sigma-Aldrich, 70% PBS). Mice were killed on day 32 when control tumors had reached about 1300 mm^3^.

### 4.6. Statistical Analysis

Statistical analysis was performed using GraphPad Prism v6 software (GraphPad Software, San Diego, CA, USA). Student’s *t* test was used to compare two groups, and one-way analysis of variance (ANOVA) was used for three or more groups; consecutive multiple comparisons were performed using Dunnett’s or Tukey’s test. *p* < 0.05 indicated statistical significance.

## 5. Conclusions

These promising results represent a starting point for further preclinical testing of these new gold (III) complexes. Additional research is needed to reveal the mechanism of action and to document the in vivo activity of the active gold (III) complexes (**C2, C3, C6**, and **C7**), in other cancer models and especially in cisplatin- or doxorubicin-resistant ones.

## Figures and Tables

**Table 1:** Half-maximal inhibitory concentrations (IC_50_) of cisplatin and new gold (III) compounds in human lung, prostate, breast, and osteosarcoma cancer cell lines. Values are mean (SD) expressed in µM.

Compound	Cell Line				
	A549	DU145	PC3	MCF-7	MG-63
Cisplatin	52.0 (4.7)	4.5 (0.4)	3.3 (0.3)	22.2 (0.2)	58.0 (0.5)
**C1**	>80	39.0 (3.5)	28.3 (2.6)	59.0 (5.3)	43.0 (3.9)
**C2**	6.1 (0.6)	2.8 (0.3)	1.5 (0.1)	2.3 (0.2)	3.8 (0.3)
**C3**	3.8 (0.3)	3.5 (0.3)	1.3 (0.1)	1.7 (0.2)	1.2 (0.1)
**C4**	25.0 (2.3)	6.4 (0.6)	8.5 (0.8)	13.0 (1.2)	12.3 (1.1)
**C5**	>80	>80	>80	65.0 (5.9)	26.0 (2.3)
**C6**	0.8 (0.1)	0.7 (0.1)	0.6 (0.1)	0.5 (0.1)	0.8 (0.1)
**C7**	1.4 (0.1)	0.8 (0.1)	0.8 (0.1)	0.6 (0.1)	0.7 (0.1)
**C8**	23.0 (2.1)	22.8 (2.1)	19.5 (1.8)	9.5 (0.9)	5.8 (0.5)

**Table 2:** Half-maximal inhibitory concentrations (IC_50_) of reference drugs and gold (III) compounds in ovarian cancer cell line A2780 and its cisplatin- and doxorubicin-resistant A2780cis clone and fold resistance (FR).

Compound	IC_50_, µM ^a^		FR (A2780cis/A2780)
	A2780	A2780cis	
Cisplatin	1.5 (0.1)	10.4 (0.9)	6.9
Doxorubicin	0.02 (0.0)	0.12 (0.0)	9.0
**C1**	23.0 (2.1)	24.0 (2.0)	1.0
**C2**	0.9 (0.1)	0.8 (0.1)	0.9
**C3**	0.4 (0.0)	0.4 (0.0)	1.1
**C4**	7.3 (0.7)	8.2 (0.7)	1.1
**C5**	15.4 (1.4)	16.2 (5.1)	1.1
**C6**	0.2 (0.0)	0.3 (0.0)	1.2
**C7**	0.4 (0.0)	0.3 (0.0)	0.9
**C8**	3.8 (0.3)	5.2 (0.5)	1.4

^a^ Mean (SD).

**Table 3:** Half-maximal inhibitory concentrations (IC_50_) of cisplatin and gold (III) compounds in cervical cancer cell line ME-180 and its cisplatin-resistant R-ME-180 clone and fold resistance (FR).

Compound	IC_50_, µM ^a^		FR (R-ME-180/ME-180)
	ME-180	R-ME-180	
Cisplatin	15.0 (1.4)	68.0 (6.1)	4.5
**C1**	70.0 (6.3)	72.0 (6.5)	1.0
**C2**	14.0 (1.3)	21.0 (1.9)	1.5
**C3**	3.0 (0.3)	3.8 (0.3)	1.3
**C4**	30.0 (2.5)	30.0 (2.7)	1.0
**C5**	>80	>80	ND
**C6**	5.3 (0.5)	4.9 (0.5)	0.9
**C7**	4.8 (0.4)	4.1 (0.4)	0.9
**C8**	15.0 (1.4)	16.0 (1.4)	1.0

ND, not determined. ^a^ Mean (SD).

## Supplementary Materials

The following are available online at https://www.mdpi.com/2072-6694/11/4/474/s1, Figure S1: Voltammograms for the interaction of **C1** with lysozyme in 0.1 M phosphate buffer (pH 6.8), Figure S2: Voltammograms for the interaction of **C2** with lysozyme in 0.1 M phosphate buffer (pH 6.8), Figure S3: Voltammograms for the interaction of **C3** with lysozyme in 0.1 M phosphate buffer (pH 6.8), Figure S4: Voltammograms for the interaction of compound **C4** with lysozyme in 0.1 M phosphate buffer (pH 6.8), Figure S5: Voltammograms for the interaction of compound **C5** with 0.5 mM tryptophan in 0.1 M phosphate buffer (pH 6.8), Figure S6: Voltammograms for the interaction of compound **C6** with 0.5 mM tryptophan in 0.1 M phosphate buffer (pH 6.8), Figure S7: Voltammograms for the interaction of compound **C7** with 0.5 mM tryptophan in 0.1 M phosphate buffer (pH 6.8); Figure S8: Voltammograms for the interaction of compound **C8** with 0.5 mM tryptophan in 0.1 M phosphate buffer (pH 6.8); Figure S9: Voltammograms for the interaction of compound **C1** with 0.5 mM guanine in 0.1 M phosphate buffer (pH 6.8), Figure S10: Voltammograms for the interaction of compound **C5** with 0.5 mM guanine in 0.1 M phosphate buffer (pH 6.8), Figure S11: Uptake of selected gold(III) compounds by PC3 cells, Figure S12: Growth inhibition curves for **C6** in PC3 prostate cancer cells and adipose-derived stromal cells (ADSCs). Table S1: Voltammetry peak potential (mV) of 100 μM compounds (**C1**–**C4**) with 1–10 μM lysozyme, Table S2: Voltammetry peak potential (mV) of 0.5 mM tryptophan with 10–100 μM compounds (**C5**–**C8**), Table S3: Voltammetry peak potential (mV) of 0.5 mM guanine with 10-100 μM compounds **C1** and **C5**.

## References

[1] Spreckelmeyer S., Orvig C., Casini A. (2014). Cellular transport mechanisms of cytotoxic metallodrugs: An overview beyond cisplatin. Molecules.

[2] Cappetta D., Rossi F., Piegari E., Quaini F., Berrino L., Urbanek K., De A.A. (2018). Doxorubicin targets multiple players: A new view of an old problem. Pharm. Res..

[3] Galluzzi L., Vitale I., Michels J., Brenner C., Szabadkai G., Harel-Bellan A., Castedo M., Kroemer G. (2014). Systems biology of cisplatin resistance: Past, present and future. Cell Death Dis..

[4] Dilruba S., Kalayda G.V. (2016). Platinum-based drugs: Past, present and future. Cancer Chemother. Pharm..

[5] Nardon C., Fregona D. (2018). Editorial: Throwing Light on Recent Advances on Metallodrugs: From Deemed Poisons to a Striking Hope for the Future. Curr. Med. Chem..

[6] Lazarevic T., Rilak A., Bugarcic Z.D. (2017). Platinum, palladium, gold and ruthenium complexes as anticancer agents: Current clinical uses, cytotoxicity studies and future perspectives. Eur. J. Med. Chem..

[7] Soldevila-Barreda J.J., Sadler P.J. (2015). Approaches to the design of catalytic metallodrugs. Curr. Opin. Chem. Biol..

[8] Casini A., Sun R.W., Ott I. (2018). Medicinal Chemistry of Gold Anticancer Metallodrugs. Met. Ions Life Sci..

[9] Bertrand B., Williams M.R.M., Bochmann M. (2018). Gold(III) Complexes for Antitumor Applications: An Overview. Chemistry.

[10] Celegato M., Borghese C., Casagrande N., Mongiat M., Kahle X.U., Paulitti A., Spina M., Colombatti A., Aldinucci D. (2015). Preclinical activity of the repurposed drug Auranofin in classical Hodgkin lymphoma. Blood.

[11] Aldinucci D., Lorenzon D., Stefani L., Giovagnini L., Colombatti A., Fregona D. (2007). Antiproliferative and apoptotic effects of two new gold(III) methylsarcosinedithiocarbamate derivatives on human acute myeloid leukemia cells in vitro. Anticancer Drugs.

[12] Milacic V., Chen D., Ronconi L., Landis-Piwowar K.R., Fregona D., Dou Q.P. (2006). A novel anticancer gold(III) dithiocarbamate compound inhibits the activity of a purified 20S proteasome and 26S proteasome in human breast cancer cell cultures and xenografts. Cancer Res..

[13] Gratteri P., Massai L., Michelucci E., Rigo R., Messori L., Cinellu M.A., Musetti C., Sissi C., Bazzicalupi C. (2015). Interactions of selected gold(III) complexes with DNA G quadruplexes. Dalton Trans..

[14] Coronnello M., Marcon G., Carotti S., Caciagli B., Mini E., Mazzei T., Orioli P., Messori L. (2000). Cytotoxicity, DNA damage, and cell cycle perturbations induced by two representative gold(III) complexes in human leukemic cells with different cisplatin sensitivity. Oncol Res..

[15] Saggioro D., Rigobello M.P., Paloschi L., Folda A., Moggach S.A., Parsons S., Ronconi L., Fregona D., Bindoli A. (2007). Gold(III)-dithiocarbamato complexes induce cancer cell death triggered by thioredoxin redox system inhibition and activation of ERK pathway. Chem. Biol..

[16] Marzano C., Ronconi L., Chiara F., Giron M.C., Faustinelli I., Cristofori P., Trevisan A., Fregona D. (2011). Gold(III)-dithiocarbamato anticancer agents: Activity, toxicology and histopathological studies in rodents. Int. J. Cancer.

[17] Cattaruzza L., Fregona D., Mongiat M., Ronconi L., Fassina A., Colombatti A., Aldinucci D. (2011). Antitumor activity of gold(III)-dithiocarbamato derivatives on prostate cancer cells and xenografts. Int. J. Cancer.

[18] Kouodom M.N., Ronconi L., Celegato M., Nardon C., Marchio L., Dou Q.P., Aldinucci D., Formaggio F., Fregona D. (2012). Toward the selective delivery of chemotherapeutics into tumor cells by targeting peptide transporters: Tailored gold-based anticancer peptidomimetics. J. Med. Chem..

[19] Nardon C., Schmitt S.M., Yang H., Zuo J., Fregona D., Dou Q.P. (2014). Gold(III)-dithiocarbamato peptidomimetics in the forefront of the targeted anticancer therapy: Preclinical studies against human breast neoplasia. PLoS ONE.

[20] Celegato M., Fregona D., Mongiat M., Ronconi L., Borghese C., Canzonieri V., Casagrande N., Nardon C., Colombatti A., Aldinucci D. (2014). Preclinical activity of multiple-target gold(III)-dithiocarbamato peptidomimetics in prostate cancer cells and xenografts. Future Med. Chem..

[21] Altaf M., Monim-ul-Mehboob M., Seliman A.A., Sohail M., Wazeer M.I., Isab A.A., Li L., Dhuna V., Bhatia G., Dhuna K. (2015). Synthesis, characterization and anticancer activity of gold(I) complexes that contain tri-tert-butylphosphine and dialkyl dithiocarbamate ligands. Eur. J. Med. Chem..

[22] Altaf M., Monom-ul-Mehboob M., Selimam A.A., Isab A.A., Dhuna V., Bhatia G., Dhuna K. (2014). Synthesis, X–ray Structures, Spectroscopic Analysis and Anticancer Activity of Novel Gold(I) Carbene Complexes. J. Organomet. Chem..

[23] Al-Jaroudi S.S., Altaf M., Al-Saadi A.A., Kawde A.N., Altuwaijri S., Ahmad S., Isab A.A. (2015). Synthesis, characterization and theoretical calculations of (1,2-diaminocyclohexane)(1,3-diaminopropane)gold(III) chloride complexes: In Vitro cytotoxic evaluations against human cancer cell lines. Biometals.

[24] Altaf M., Monim-ul-Mehboob M., Kawde A.N., Corona G., Larcher R., Ogasawara M., Casagrande N., Celegato M., Borghese C., Siddik Z.H. (2017). New bipyridine gold(III) dithiocarbamate-containing complexes exerted a potent anticancer activity against cisplatin-resistant cancer cells independent of p53 status. Oncotarget.

[25] Scalcon V., Bindoli A., Rigobello M.P. (2018). Significance of the mitochondrial thioredoxin reductase in cancer cells: An update on role, targets and inhibitors. Free Radic. Biol. Med..

[26] Zhang J., Li X., Han X., Liu R., Fang J. (2017). Targeting the Thioredoxin System for Cancer Therapy. Trends Pharm. Sci..

[27] Shan W., Zhong W., Zhao R., Oberley T.D. (2010). Thioredoxin 1 as a subcellular biomarker of redox imbalance in human prostate cancer progression. Free Radic. Biol. Med..

[28] Yamada M., Tomida A., Yoshikawa H., Taketani Y., Tsuruo T. (1996). Increased expression of thioredoxin/adult T-cell leukemia-derived factor in cisplatin-resistant human cancer cell lines. Clin. Cancer Res..

[29] Marzano C., Gandin V., Folda A., Scutari G., Bindoli A., Rigobello M.P. (2007). Inhibition of thioredoxin reductase by auranofin induces apoptosis in cisplatin-resistant human ovarian cancer cells. Free Radic. Biol. Med..

[30] Baumann K. (2014). Protein metabolism: How the proteasome adapts to stress. Nat. Rev. Mol. Cell Biol..

[31] Manasanch E.E., Orlowski R.Z. (2017). Proteasome inhibitors in cancer therapy. Nat. Rev. Clin. Oncol..

[32] Tomasello M.F., Nardon C., Lanza V., Di N.G., Pettenuzzo N., Salmaso S., Milardi D., Caliceti P., Pappalardo G., Fregona D. (2017). New comprehensive studies of a gold(III) Dithiocarbamate complex with proven anticancer properties: Aqueous dissolution with cyclodextrins, pharmacokinetics and upstream inhibition of the ubiquitin-proteasome pathway. Eur. J. Med. Chem..

[33] Quero J., Cabello S., Fuertes T., Marmol I., Laplaza R., Polo V., Gimeno M.C., Rodriguez-Yoldi M.J., Cerrada E. (2018). Proteasome versus Thioredoxin Reductase Competition as Possible Biological Targets in Antitumor Mixed Thiolate-Dithiocarbamate Gold(III) Complexes. Inorg. Chem..

[34] Ritch C., Cookson M. (2018). Recent trends in the management of advanced prostate cancer. F1000Reseach.

[35] Nobili S., Mini E., Landini I., Gabbiani C., Casini A., Messori L. (2010). Gold compounds as anticancer agents: Chemistry, cellular pharmacology, and preclinical studies. Med. Res. Rev..

[36] Micale N., Schirmeister T., Ettari R., Cinellu M.A., Maiore L., Serratrice M., Gabbiani C., Massai L., Messori L. (2014). Selected cytotoxic gold compounds cause significant inhibition of 20S proteasome catalytic activities. J. Inorg. Biochem..

[37] Chou T.C., Talalay P. (1984). Quantitative analysis of dose-effect relationships: The combined effects of multiple drugs or enzyme inhibitors. Adv. Enzym. Regul..

