# Supplementary material for: Potent In Vitro and In Vivo Anticancer Activity of New Bipyridine and Bipyrimidine Gold (III) Dithiocarbamate Derivatives
Source: Cancers (Basel). 2019 Apr 4;11(4):474. doi: 10.3390/cancers11040474 (PMC6521029; doi:10.3390/cancers11040474)
Supplement: Supplementary file 1 [file cancers-11-00474-s001.pdf]

Supplementary Materials: Potent in vitro and in vivo anticancer activity of new bipyridine and bipyrimidine gold(III)-dithiocarbamate derivatives

Muhammad Altaf, Naike Casagrande, Elena Mariotto, Nadeem Baig, Abdel-Nasser Kawde, Giuseppe Corona, Roberto Larcher, Cinzia Borghese, Claudia Pavan, Adam A. Seliman, Donatella Aldinucci and Anvarhusein A. Isab

Supplementary Materials and Methods

Reagents

Sodium tetrachloroaurate(III) dihydrate, sodium dimethyldithiocarbamate hydrate, sodium diethyldithiocarbamate trihydrate, sodium dibenzyldithiocarbamate hydrate, 2,2'-bipyrimidine, 2,2'-bipyridine-3,3'-diol, disodium hydrogen phosphate, sodium dihydrogen phosphate, tryptophan, lysozyme, 98% guanine, and 99.8% ethanol were obtained from Sigma-Aldrich. Anhydrous 99.8% dichloromethane were purchased from Merck and used without further purification. Double distilled water used only for electrochemical measurements was from an Aquatron A4000D water still (Stuart). All reactions were carried out at ambient room temperature.

Synthesis of gold(III) compounds (C1-C8)

[Au(BPYH)(Cl)$_2$]Cl (C1) was synthesized by combining 0.5 mM Na[AuCl$_4$].2H$_2$O (200 mg in 3 mL H$_2$O) and 0.5 mM 2,2′-bipyridine-3,3′-diol (94 mg in 15 mL of ethanol:dichloromethane (3:1)) and stirred for 3 h. The solution was filtered and kept in an undisturbed area for 3 days. Black cubic crystals appeared. The black precipitate was collected by filtration, washed with distilled water (3 × 10 mL) and dried under vacuum.

[Au(BPYH)(DMDTC)]Cl$_2$ (C2) was synthesized stepwise. First, 0.5 mM Na[AuCl$_4$].2H$_2$O (200 mg in 3 mL distilled water) and 0.5 mM 2,2′-bipyridine-3,3′-diol (94.0 mg in 15 mL ethanol:dichloromethane (3:1)) were added simultaneously to 20 mL of 99.8% ethanol, and the mixture was stirred for 3 h, generating a pale yellow, turbid solution. Second, 0.5 mM sodium dimethyldithiocarbamate hydrate (71.6 mg in 10 mL distilled water) was added dropwise and the mixture was stirred for an additional 1 h. The product light-yellow precipitate was collected by filtration, washed with distilled water (3 × 10 mL), and dried under vacuum.

[Au(BPYH)(DEDTC)]Cl$_2$ (C3) was synthesized stepwise. First, 0.5 mM Na[AuCl$_4$].2H$_2$O (200 mg in 3 mL H$_2$O) and 0.5 mM 2,2′-bipyridine-3,3′-diol (94.0 mg in 15 mL ethanol:dichloromethane (3:1)) were added simultaneously to 20 mL of 99.8% ethanol, and the mixture was stirred for 3 h, generating a yellow turbid solution. Second, 0.5 mM sodium diethyldithiocarbamate trihydrate (112.6 mg in 10 mL distilled water) was added dropwise and the mixture was stirred for an additional 1 h. The obtained yellow precipitate was collected by filtration, washed with distilled water (3 × 10 mL) and dried under vacuum. The final product was a yellow crystalline powder.

[Au(BPYH)(DBDTC)]Cl$_2$ (C4) was synthesized stepwise. First, 0.5 mM 2,2′-bipyridine-3,3′-diol (94.0 mg in 15 mL ethanol:dichloromethane (3:1)) was combined with 0.5 mM Na[AuCl$_4$].2H$_2$O (200 mg in 3 mL distilled water) and the mixture was stirred for 3 h, generating an orange turbid solution. Second, 0.5 mM sodium dibenzyldithiocarbamate hydrate (148 mg in 10 mL 99.8% ethanol) was added dropwise and the mixture was stirred for an additional 1 h. The orange precipitate was collected by filtration, washed with distilled water (3 × 10 mL) and dried under vacuum. The final product was an orange crystalline powder.

[Au$_2$(BPM)(Cl)$_4$]Cl$_2$ (C5) was synthesized by combining 0.5 mM 2,2′-bipyrimidine (79 mg in 20 mL 99.8% ethanol) and 1.0 mM Na[AuCl$_4$].2H$_2$O (397.8 mg in 10 mL distilled water). The mixture was stirred for 3 h. The yellow precipitate was collected by filtration, washed with distilled water (3 × 10 mL), and dried under vacuum. ′

[Au$_2$(BPM)(DMDTC)$_2$]Cl$_4$ (C6) was synthesized stepwise. First, 0.5 mM 2,2′-bipyrimidine (79 mg in 20 mL 99.8% ethanol) was combined with 1.0 mM Na[AuCl$_4$].2H$_2$O (397.8 mg in 3 mL distilled water). The mixture was stirred for 3 h, generating a bright yellow, turbid solution. Then, 1.0 mM sodium

dimethyldithiocarbamate hydrate (143.2 mg in 20 mL distilled water) was slowly added, and the reaction mixture was stirred for 1 h. The product appeared as a pale yellow precipitate. The precipitate was collected by filtration, washed with distilled water (3 × 10 mL) and dried under vacuum.

$[Au_2(BPM)(DEDTC)_2]Cl_4$ (C7) was synthesized stepwise. First, 0.5 mM 2,2′-bipyrimidine (79 mg in 20 mL 99.8% ethanol) was combined with 1.0 mM $Na[AuCl_4].2H_2O$ (397.8 mg in 3.0 mL distilled water). The mixture was stirred for 3 h, generating a bright yellow, turbid solution. Then, 1.0 mM sodium diethyldithiocarbamate hydrate (226 mg in 20 mL distilled water) was slowly added, and the reaction mixture was stirred for 1 h. The product appeared as a dark yellow precipitate; it was collected by filtration, washed with distilled water (3 × 10 mL), and dried under vacuum for 72 hours.

$[Au_2(BPM)(DBDTC)_2]Cl_4$ (C8) was synthesized stepwise. First, 0.5 mM 2,2′-bipyrimidine (79 mg in 20 mL 99.8% ethanol) was combined with 1.0 mM $Na[AuCl_4].2H_2O$ (397.8 mg in 3 mL distilled water). The mixture was stirred for 3 h, generating a yellow, turbid solution. Then, 1.0 mM sodium diethyldithiocarbamate hydrate (295.4 mg in 20 mL distilled water) was slowly added, and the reaction mixture was stirred for 1 h. The product appeared as a yellowish green precipitate; it was collected by filtration, washed with distilled water (3 × 10 mL), and dried under vacuum for 72 h.

Chemical characterization of gold(III) compounds

Due to the poor solubility of the gold(III) compounds in water, they were dissolved in 99.8% ethanol. The pH of buffers was monitored on a Accumet XL50 pH meter. A GR-2000 electrical balance was used to weigh the various chemicals. Electrochemical measurements for cyclic voltammetry and square wave voltammetry were performed using Autolab instruments (Metrohm; Netherlands). The electrochemical workstation had three electrodes (from CH Instruments): a glassy carbon electrode (GCE) as the working electrode, platinum as the counter electrode, and Ag/AgCl as the reference electrode (in saturated KCl). The GCE was polished as a mirror-like surface with alumina slurry on a synthetic cloth before every electrochemical analysis. Square wave voltammetry and cyclic voltammetry were scanned from 0 to 1.3 V for the various analyses. Elemental analyses of gold(III) compounds **(C1-C8)** were performed on PerkinElmer Series 11 (CHNS/O), Analyzer 2400.

The solid state FTIR spectra of sodium dimethyldithiocarbamate hydrate, sodium diethyldithiocarbamate trihydrate, and sodium dibenzyldithiocarbamate hydrate (free ligands) and their corresponding gold(III) complexes were recorded on a PerkinElmer FTIR 180 spectrophotometer or NICOLET 6700 FTIR using potassium bromide (KBr) pellets over the range 4000–400 cm^{-1}. ^{1}H and ^{13}C NMR spectra were recorded on a LAMBDA 500 spectrophotometer operating at 500.01 and 125.65 MHz respectively, corresponding to a magnetic field of 11.74 T. Tetramethylsilane was used as an internal standard for ^{1}H and ^{13}C. The ^{13}C NMR spectra were obtained with ^{1}H broadband decoupling, and the spectral conditions were: 32 k data points, 0.967 s acquisition time, 1.00 s pulse delay and 45 g pulse angle.

Gold (III) compound interactions with lysozyme, tryptophan and guanine

The electrochemical investigation of the interactions between the gold(III) compounds (**C1-C8**) and lysozyme, tryptophan and guanine was performed using the Autolab instrument described above, with a three-electrode system (CH Instruments): platinum wire counter electrode (CHI115), Ag/AgCl reference electrode (in 3 M KCl, CHI111) and glassy carbon working electrode (CHI112) inserted into a 5.0 ml glass cell. Solutions of 1 mM lysozyme, 5 mM tryptophan and 5 mM guanine were prepared in double distilled water, and the experiment was performed in 0.1 M phosphate buffer at pH 6.8.

Cellular uptake of gold (III) compounds

PC3 cells (1 × 10^6 cells seeded in 100 × 20 culture dishes) were treated for 2 h in duplicate with 3 μM **C4**, **C5**, **C6** or **C7** in complete culture medium. After treatment, monolayers were washed with ice-cold PBS four times, and the cells were detached with trypsin-EDTA and washed three times with ice-cold PBS by centrifugation. The cell pellet was solubilized in 700 μL of HNO_3-HCl solution (1:3 molar ratio) for 2 h at 100 °C, and then diluted with 4 mL water. Samples were analyzed for gold on an Agilent 7500 inductively

coupled plasma mass spectrometer (ICP-MS). Results were expressed as ng gold/10^6 cells. The experiment was performed a total of two times and the results were expressed as mean and SD.

Growth inhibition curves for C6 in adipose-derived stromal cells

Human adipose-derived stromal cells (ADSCs) were from Lonza (Verviers, Belgium). ADSCs were maintained in MSGM bullet kit (Lonza) and experiments were performed in DMEM (Cambrex Bio Science, Milan, Italy) supplemented with 10 % FBS. To evaluate effects of **C6**, ADSCs were seeded in 96-well flat-bottomed microplates (5.0 × 10^3 cells in 100 μL per well) and incubated for 24 h (to allow cell adhesion) before drug testing. The medium was removed and replaced with fresh medium containing **C6** at increasing concentrations (from 0.1 to 1 μM). Cells were incubated at 37 °C for 72 h. Each treatment was performed in triplicate. Cell growth was measured using the MTT assay.

Supplementary Results

Analytical data of gold(III) compounds

[Au(BPYH)(Cl)$_2$]Cl (**C1**). Yield: 83.09% (219.98 mg). FT-IR (KBr, υ_{max}, cm^{-1}): 3470 (b), 3071 (w), 1647 (m), 1584 (s), 1459 (s), 1269 (m), 1134 (s), 1012 (m), 916 (w), 790 (s), 565 (m), 508 (m). ^{1}H NMR (500 MHz, DMSO-d_6): δ = 7.57, 8.25 and 8.77 (3H, 2 × CH, BPYH). ^{13}C NMR (125.65 MHz, DMSO-d_6): δ = 125.81, 128.31, 136.68, 138.63 and 155.31 (BPYH). Anal. calc. for $C_{10}H_7Cl_2N_2O_2Au$ (456); C, 26.38; H, 1.55; N, 6.16; Found: C, 26.42; H, 1.53; N, 6.18%.

[Au(BPYH)(DMDTC)]Cl$_2$ (C2). Yield: 83.09% (219.98 mg). FT-IR (KBr, υ_{max}, cm^{-1}): 3437 (b), 3055 (w), 2921 (w); 1576 (s), 1484 (s), 1299 (m), 1111 (w), 1060 (m), 989 (w), 795 (s), 580 (m). ^{1}H NMR (500 MHz, DMSO-d_6): δ = 2.49 (6H, 2 × CH_3), 7.51, 8.18 and 8.63 (2H, 2 × CH, BPYH). ^{13}C NMR (125.65 MHz, DMSO-d_6): δ = 39.38 (CH_3), 126.20, 128.30, 135.49, 138.74 and 155.33 (2,2'-BPYH), 193.84 (NC = S). Anal. calc. for $C_{13}H_{13}Cl_1N_3O_2S_2Au$ (539.81): C, 28.92; H, 2.43; N, 7.79; S, 11.88%. Found: C, 28.97; H, 2.39; N, 7.81; S, 11.91%.

[Au(BPYH)(DEDTC)]Cl$_2$ (C3). Yield: 86.87% (166.87 mg). FT-IR (KBr, υ_{max}, cm^{-1}): 3443 (b), 3047 (w), 2928 (w), 1572 (s), 1490 (s), 1235 (m), 1155 (w), 1063 (m), 876 (m), 796 (s), 548 (m). ^{1}H NMR (500 MHz, DMSO-d_6): δ = 2.49 (6H, 2 × CH_3), 3.75 (4H, 2 × CH_2), 7.49, 8.18 and 8.62 (2H, 2 × CH, BPYH). ^{13}C NMR (125.65 MHz, DMSO-d_6): δ = 12.11 (CH_3), 46.52 (CH_2), 125.78, 128.21, 136.74, 138.79 and 155.33 (2,2′-BPYH), 195.11 (NC = S). Anal. calc. for $C_{15}H_{17}Cl_1N_3O_2S_2Au$ (567.86): C, 31.73; H, 3.02; N, 7.40; S, 11.29%. Found: C, 31.76; H, 2.99; N, 7.43; S, 11.31%.

[Au(BPYH)(DBDTC)]Cl$_2$ (C4). Yield: 85.55% (226.13 mg). FT-IR (KBr, υ_{max}, cm^{-1}): 3444 (b), 3021 (w), 2925 (w); 1532 (s), 1434 (s), 1223 (s), 1117 (m), 1068 (m), 982 (m), 796 (s), 549 (m). ^{1}H NMR (500 MHz, DMSO-d_6): δ = 5.78 (4H, 2 × CH_2), 8.10 (10H, 2 × C_6H_5), 8.18, 8.27 and 8.93 (2H, 2 × CH, BPYH). ^{13}C NMR (125.65 MHz, DMSO-d_6): δ = 55.01 (CH_2), 125.67, 128.93, 138.78 and 155.35 (BPYH), 128.21– 132.49 (C_6H_5), 199.10 (NC = S). Anal. calc. for $C_{25}H_{21}Cl_1N_3O_2S_2Au$ (692.00): C, 43.39; H, 3.06; N, 6.07; S, 9.27%. Found: C, 43.41; H, 3.05; N, 6.09; S, 9.31%.

[Au$_2$(BPM)(Cl)$_4$]Cl$_2$ (C5). Yield: 80.09% (187.27 mg). FT-IR (KBr, υ_{max}, cm^{-1}): 3073 (m), 1577 (s), 1406 (s), 1226 (m), 1113 (m), 1023 (m), 821 (m), 570 (m). ^{1}H NMR (500 MHz, DMSO-d_6): δ = 7.35 and 8.72 (4H, 4 × CH and 2H, 2 × CH, BPM). ^{13}C NMR (125.65 MHz, DMSO-d_6): δ = 121.99, 157.89 and 161.84 (BPM). Anal. calc. for $C_8H_6Cl_6N_4Au_2$ (764.81): C, 12.56; H, 0.79; N, 7.33%. Found: C, 12.59; H, 0.78; N, 7.35%.

[Au$_2$(BPM)(DMDTC)$_2$]Cl$_4$ (C6). Yield: 80.09% (187.27 mg). FT-IR (KBr, υ_{max}, cm^{-1}): 3057 (w), 2925 (w), 1578 (s), 1402 (s), 1238 (m), 1163 (m), 1046 (m), 967 (w), 876 (w), 559 (m). ^{1}H NMR (500 MHz, DMSO-d_6): δ = 2.35 (6H, 2 × CH_3), 7.35 and 8.70 (4H, 4 × CH and 2H, 2× CH, BPM). ^{13}C NMR (125.65 MHz, DMSO-d_6): δ = 40.29 (CH_3), 121.28, 135.33, 140.27 and 147.80 (2,2'-BPM), 193.87 (NC = S). Anal. calc. for $C_{14}H_{18}Cl_4N_6S_4Au_2$ (934.34): C, 18.00; H, 1.94; N, 8.99; S, 13.73%. Found: C, 18.05; H, 1.91; N, 9.03; S, 13.77%.

[Au$_2$(BPM)(DEDTC)$_2$]Cl$_4$ (C7). Yield: 88.51% (343.9 mg). FT-IR (KBr, υ_{max}, cm^{-1}): 3055 (w), 2978 (w), 2930 (w), 1552 (s), 1463 (s), 1351 (m), 1286 (s), 1195 (m), 1089 (m), 994 (m), 846 (m), 584 (m). ^{1}H NMR (500 MHz, DMSO-d_6): δ = 2.43 (6H, 2 × CH_3), 3.85 (4H, 2 × CH_2), δ = 7.33 and 8.71 (4H, 4 × CH and 2H, 2 × CH, BPM). ^{13}C NMR (125.65 MHz, DMSO-d_6): δ = 12.13 (CH_3), 46.58 (CH_2), 120.55, 156.63 and 161.75 (BPM), 193.89 (NC = S). Anal. calc. for $C_{18}H_{26}Cl_4N_6S_4Au_2$ (990.44): C, 21.83; H, 2.65; N, 8.49; S, 12.95%. Found: C, 21.88; H, 2.61; N, 8.55; S, 13.07%.

[Au_2(BPM)(DBDTC)$_2$]Cl_4 (C8). Yield: 78.01% (256.6 mg). FT-IR (KBr, υ_{max}, cm^{-1}): 3051 (w), 2972 (w), 2922 (m), 1573 (s), 1471 (s), 1355 (m), 1234 (s), 1133 (m), 1047 (m), 980 (s), 553 (m). ^{1}H NMR (500 MHz, DMSO-d_6): δ = 5.02 (4H, 2 × CH_2), 7.37 (10H, 2 × C_6H_5), 7.33 and 8.75 (4H, 4 × CH and 2H, 2 × CH, BPM). ^{13}C NMR (125.65 MHz, DMSO-d_6): δ = 55.37 (CH_2), 120.81, 156.23 and 161.90 (BPM), 128.17–132.50 (C_6H_5), 199.14 (NC = S). Anal. calc. for $C_{38}H_{34}Cl_4N_6S_4Au_2$ (1238.72): C, 36.85; H, 2.77; N, 6.78; S, 10.35%. Found: C, 36.88; H, 2.73; N, 6.80; S, 10.37%.

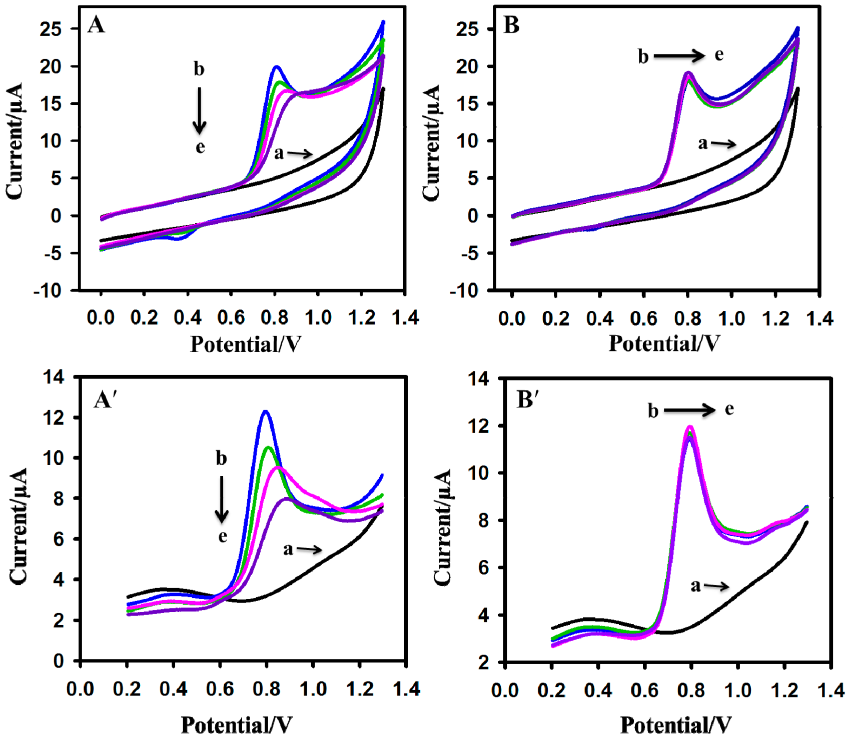

Figure S1. Voltammograms for the interaction of **C1** with lysozyme in 0.1 M phosphate buffer (pH 6.8). (**A**) Cyclic voltammetry of 100 μM **C1** with varying concentrations of lysozyme: (a) buffer blank; (b) **C1** alone; (c) **C1** and 1 μM lysozyme; (d) **C1** and 4 μM lysozyme; and (e) **C1** and 10 μM lysozyme. (**A′**) Square-wave voltammetry of 100 μM **C1** with various concentrations of lysozyme as in (A). (**B**) Cyclic voltammetry of 100 μM **C1** in control experiments with varying volumes of double-distilled water: (a) buffer blank; (b) 0 μL; (c) 3 μL; (d) 12 μL; and (e) 30 μL. (**B′**) Square-wave voltammetry of 100 μM **C1** in control experiments with varying volumes of double-distilled water as in (B).

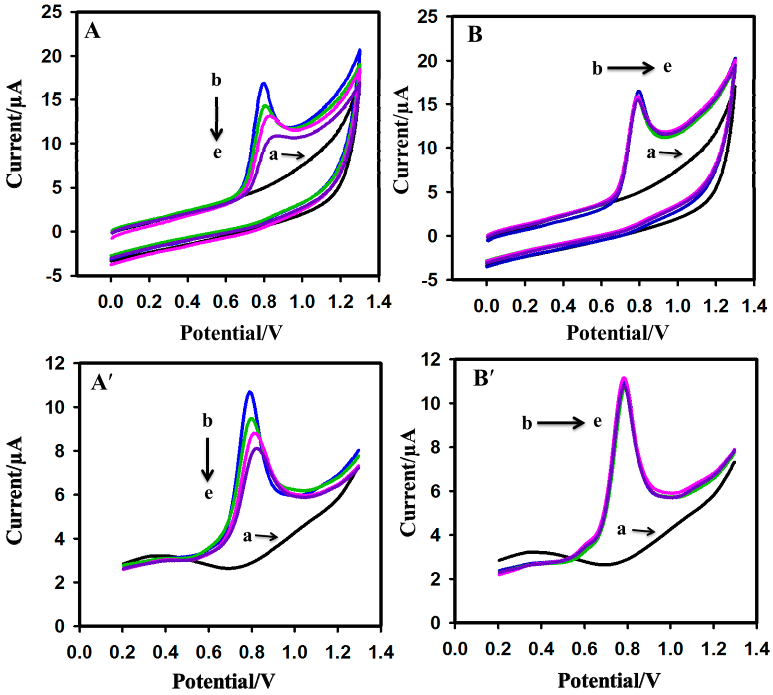

Figure S2. Voltammograms for the interaction of **C2** with lysozyme in 0.1 M phosphate buffer (pH 6.8). (**A**) Cyclic voltammetry of 100 µM **C2** with varying concentrations of lysozyme: (a) buffer blank; (b) **C2** alone; (c) **C2** and 1 µM lysozyme; (d) **C2** and 4 µM lysozyme; and (e) **C2** and 10 µM lysozyme. (**A′**) Square-wave voltammetry of 100 µM **C2** with various concentrations of lysozyme as in (A). (**B**) Cyclic voltammetry of 100 µM **C2** in control experiments with varying volumes of double-distilled water: (a) buffer blank; (b) 0 µL; (c) 3 µL; (d) 12 µL; and (e) 30 µL. (**B′**) Square-wave voltammetry of 100 µM **C2** in control experiments with varying volumes of double-distilled water as in (B).

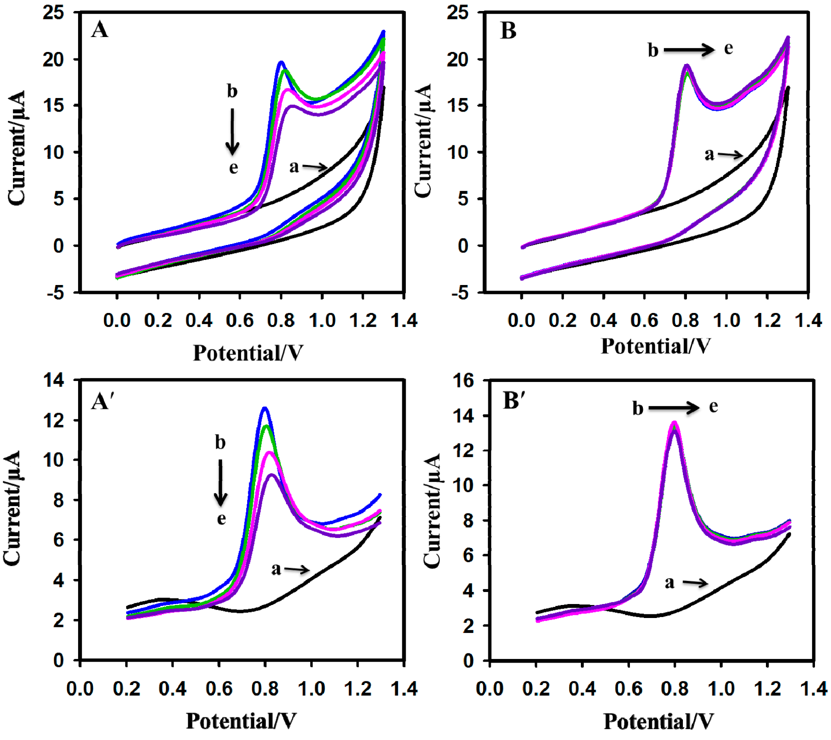

Figure S3. Voltammograms for the interaction of **C3** with lysozyme in 0.1 M phosphate buffer (pH 6.8). (**A**) Cyclic voltammetry of 100 µM **C3** with varying concentrations of lysozyme: (a) buffer blank; (b) **C3** alone; (c)

C3 and 1 μM lysozyme; (d) **C3** and 4 μM lysozyme; and (e) **C3** and 10 μM lysozyme. (**A′**) Square-wave voltammetry of 100 μM **C3** with various concentrations of lysozyme as in (A). (**B**) Cyclic voltammetry of 100 μM **C3** in control experiments with varying volumes of double-distilled water: (a) buffer blank; (b) 0 μL; (c) 3 μL; (d) 12 μL; and (e) 30 μL. (**B′**) Square-wave voltammetry of 100 μM **C3** in control experiments with varying volumes of double-distilled water as in (B).

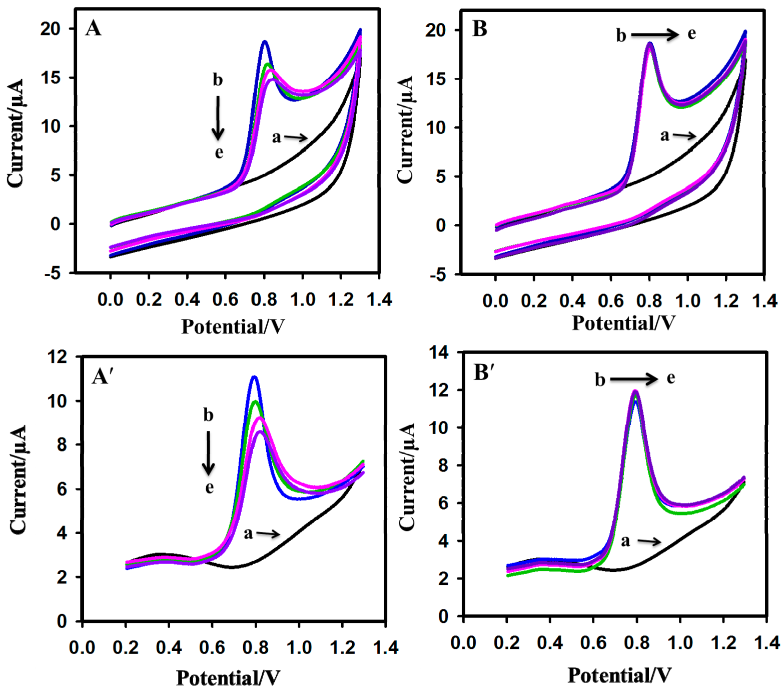

Figure S4. Voltammograms for the interaction of compound **C4** with lysozyme in 0.1 M phosphate buffer (pH 6.8). (**A**) Cyclic voltammetry of 100 μM **C4** with varying concentrations of lysozyme: (a) buffer blank; (b) **C4** alone; (c) **C4** and 1 μM lysozyme; (d) **C4** and 4 μM lysozyme; and (e) **C4** and 10 μM lysozyme. (**A′**) Square-wave voltammetry of 100 μM **C4** with various concentrations of lysozyme as in (A). (**B**) Cyclic voltammetry of 100 μM **C4** in control experiments with varying volumes of double-distilled water: (a) buffer blank; (b) 0 μL; (c) 3 μL; (d) 12 μL; and (e) 30 μL. (**B′**) Square-wave voltammetry of 100 μM **C4** in control experiments with varying volumes of double-distilled water as in (B).

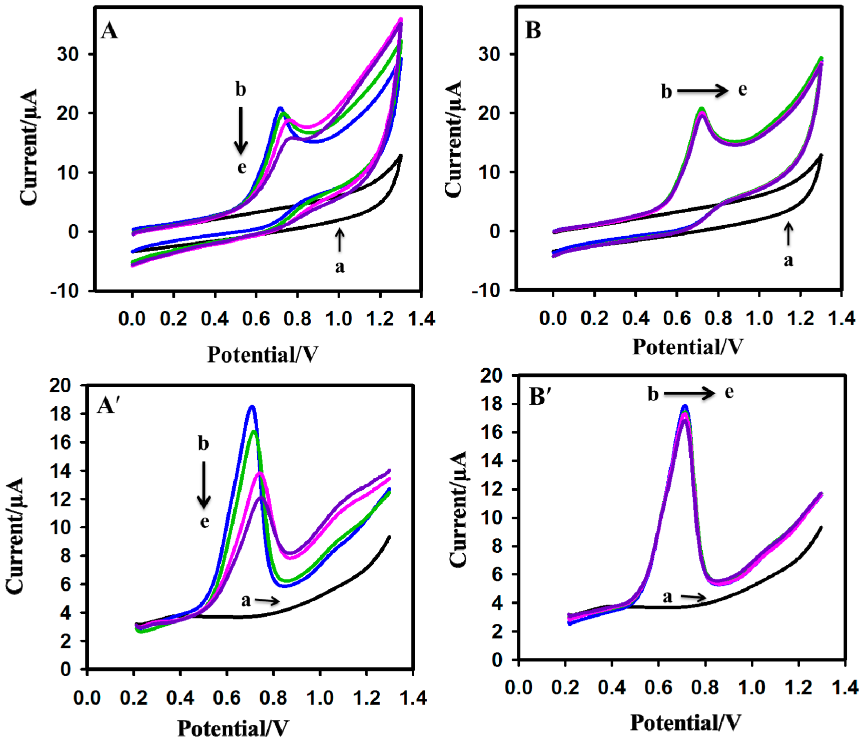

Figure S5. Voltammograms for the interaction of compound **C5** with 0.5 mM tryptophan in 0.1 M phosphate buffer (pH 6.8). (**A**) Cyclic voltammetry of 0.5 mM tryptophan with varying concentrations of **C5**: (a) buffer blank; (b) tryptophan alone; (c) tryptophan and 10 μM **C5**; (d) tryptophan and 40 μM **C5**; and (e) tryptophan and 100 μM **C5**. (**A′**) Square-wave voltammetry of 0.5 mM tryptophan with varying concentrations of **C5** as in (A). (**B**) Cyclic voltammetry of 0.5 mM tryptophan in control experiments with varying volumes of ethanol: (a) buffer blank; (b) 0 μL; (c) 15 μL; (d) 60 μL; and (e) 150 μL. (**B′**) Square-wave voltammetry of 100 μM **C5** in control experiments with varying volumes of ethanol as in (B).

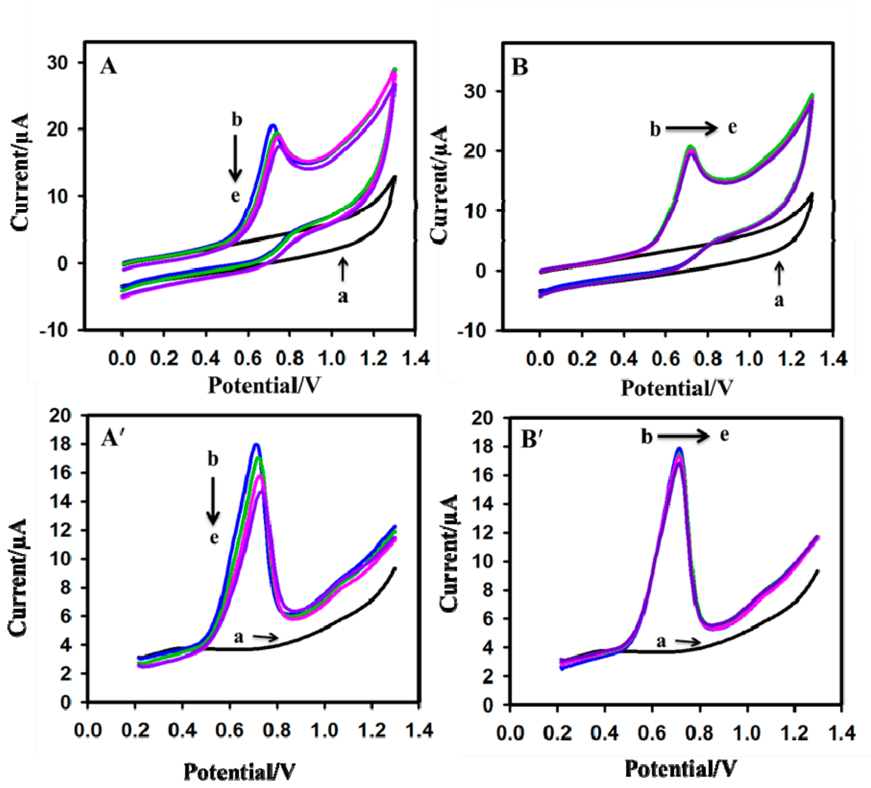

Figure S6. Voltammograms for the interaction of compound **C6** with 0.5 mM tryptophan in 0.1 M phosphate buffer (pH 6.8). (**A**) Cyclic voltammetry of 0.5 mM tryptophan with varying concentrations of **C6**: (a) buffer blank; (b) tryptophan alone; (c) tryptophan and 10 µM **C6**; (d) tryptophan and 40 µM **C6**; and (e) tryptophan and 100 µM **C6**. (**A′**) Square-wave voltammetry of 0.5 mM tryptophan with varying concentrations of **C6** as in (A). (**B**) Cyclic voltammetry of 0.5 mM tryptophan in control experiments with varying volumes of ethanol: (a) buffer blank; (b) 0 µL; (c) 15 µL; (d) 60 µL; and (e) 150 µL. (**B′**) Square-wave voltammetry of 100 µM **C6** in control experiments with varying volumes of ethanol as in (B).

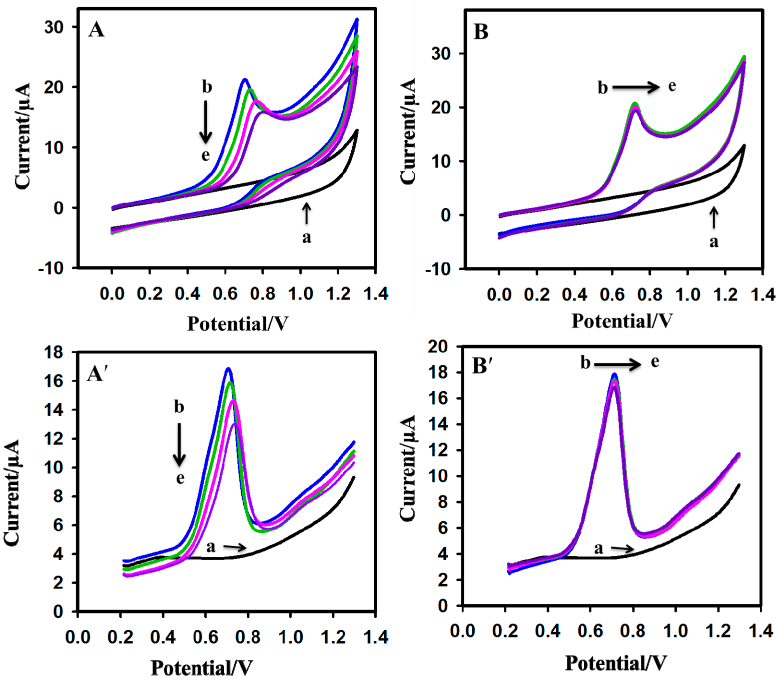

Figure S7. Voltammograms for the interaction of compound **C7** with 0.5 mM tryptophan in 0.1 M phosphate buffer (pH 6.8). (**A**) Cyclic voltammetry of 0.5 mM tryptophan with varying concentrations of **C7**: (a) buffer blank; (b) tryptophan alone; (c) tryptophan and 10 µM **C7**; (d) tryptophan and 40 µM **C7**; and (e) tryptophan and 100 µM **C7**. (**A′**) Square-wave voltammetry of 0.5 mM tryptophan with varying concentrations of **C7** as in (A). (**B**) Cyclic voltammetry of 0.5 mM tryptophan in control experiments with varying volumes of ethanol: (a) buffer blank; (b) 0 µL; (c) 15 µL; (d) 60 µL; and (e) 150 µL. (**B′**) Square-wave voltammetry of 100 µM **C7** in control experiments with varying volumes of ethanol as in (B).

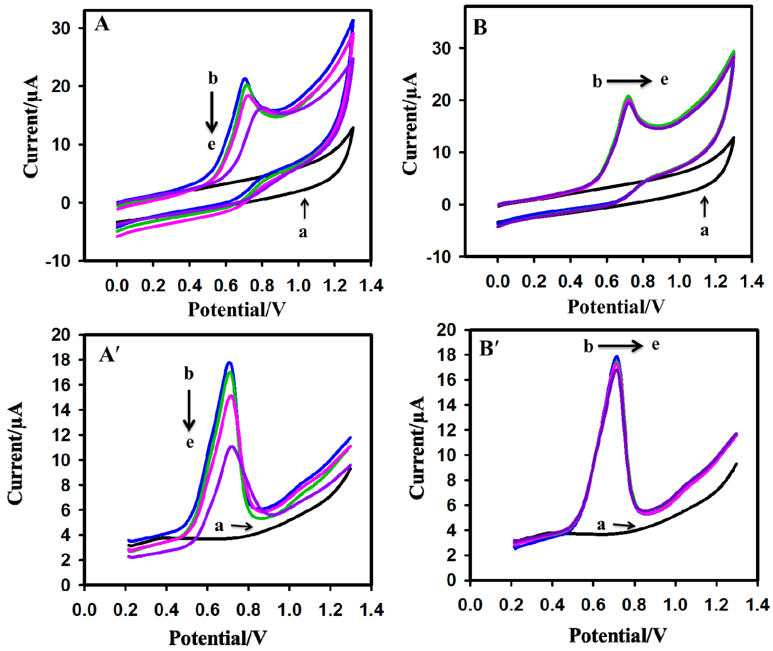

Figure S8. Voltammograms for the interaction of compound **C8** with 0.5 mM tryptophan in 0.1 M phosphate buffer (pH 6.8). (**A**) Cyclic voltammetry of 0.5 mM tryptophan with varying concentrations of **C8**: (a) buffer blank; (b) tryptophan alone; (c) tryptophan and 10 µM **C8**; (d) tryptophan and 40 µM **C8**; and (e) tryptophan and 100 µM **C8**. (**A′**) Square-wave voltammetry of 0.5 mM tryptophan with varying concentrations of **C8** as in (A). (**B**) Cyclic voltammetry of 0.5 mM tryptophan in control experiments with varying volumes of ethanol: (a) buffer blank; (b) 0 µL; (c) 15 µL; (d) 60 µL; and (e) 150 µL. (**B′**) Square-wave voltammetry of 100 µM **C5** in control experiments with varying volumes of ethanol as in (B).

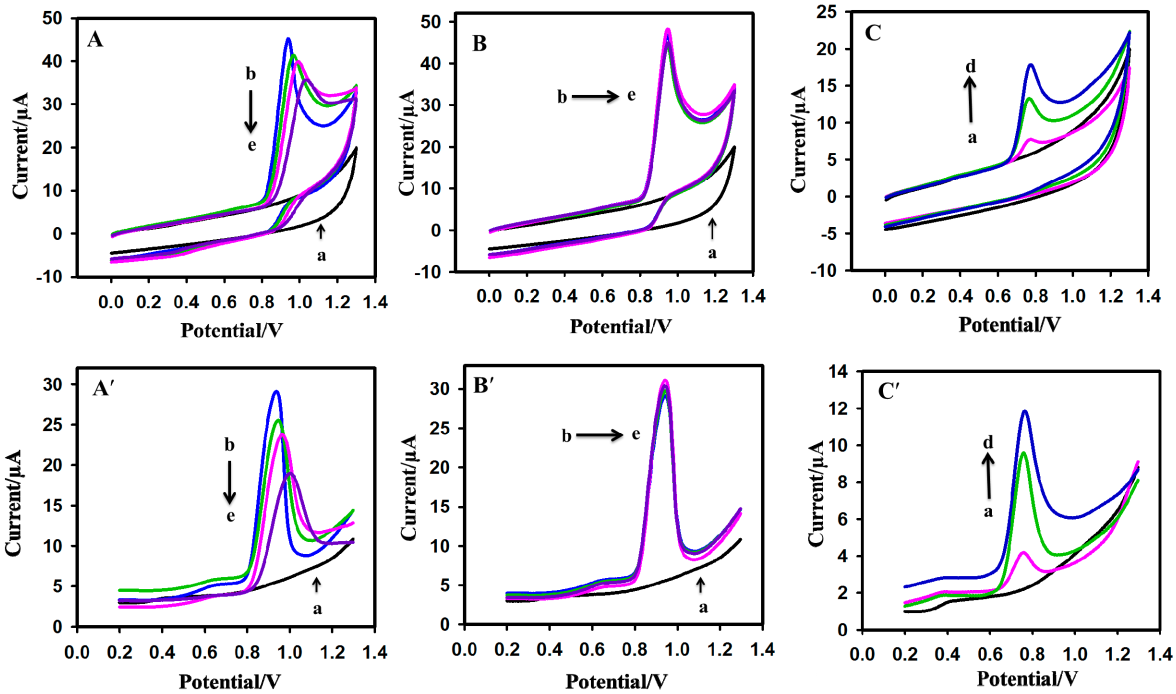

Figure S9. Voltammograms for the interaction of compound **C1** with 0.5 mM guanine in 0.1 M phosphate buffer (pH 6.8). (**A**) Cyclic voltammetry of 0.5 mM guanine with varying concentrations of **C1:** (a) buffer blank; (b) guanine alone; (c) guanine and 10 µM **C1;** (d) guanine and 40 µM **C1;** and (e) guanine and 100 µM **C1**. (**A′**) Square-wave voltammetry of 0.5 mM guanine with varying concentrations of **C1** as in (A). (**B**) Cyclic

voltammetry of 0.5 mM guanine in control experiments with varying volumes of ethanol: (a) buffer blank; (b) 0 µL; (c) 15 µL; (d) 60 µL; and (e) 150 µL. (**B′**) Square-wave voltammetry of 0.5 mM guanine in control experiments with varying volumes of ethanol: (a) buffer blank; (b) 0 µL; (c) 15 µL; (d) 60 µL; and (e) 150 µL. (**C**) Cyclic voltammetry of **C1** alone at: (a) 0 µM; (b) 10 µM; (c) 40 µM; and (d) 100 µM. (**C′**) Square-wave voltammetry of **C1** alone at: (a) 0 µM; (b) 10 µM; (c) 40 µM; and (d) 100 µM.

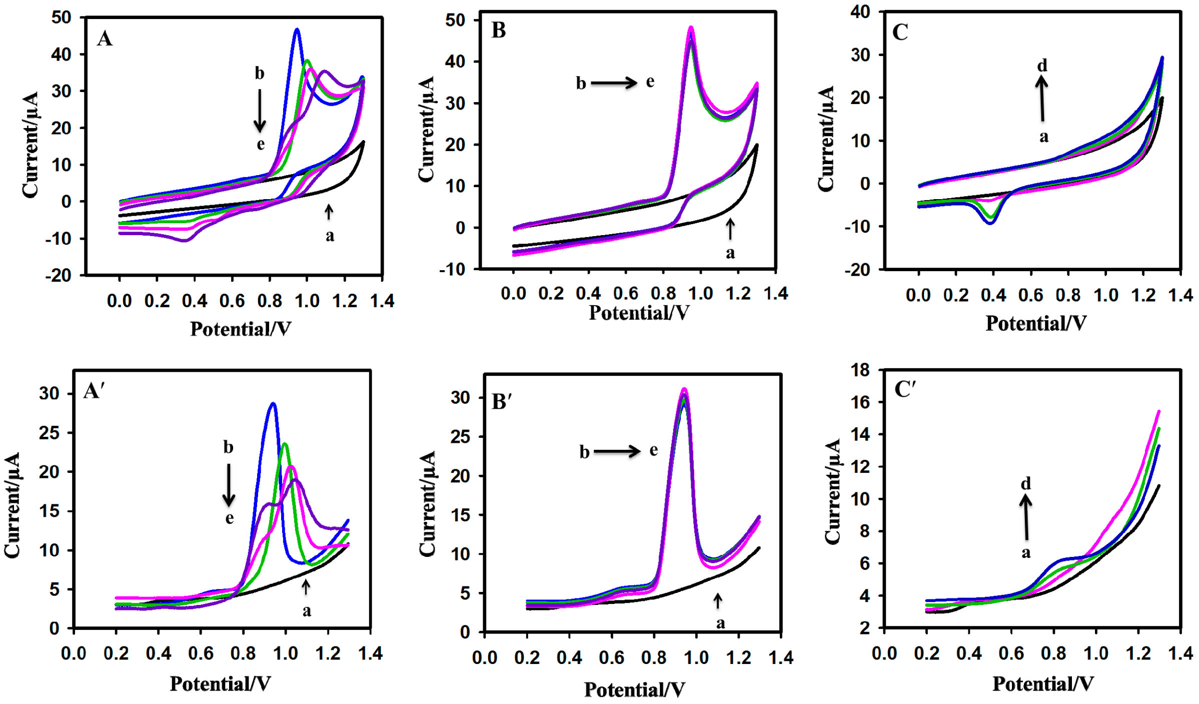

Figure S10. Voltammograms for the interaction of compound **C5** with 0.5 mM guanine in 0.1 M phosphate buffer (pH 6.8). (A) Cyclic voltammetry of 0.5 mM guanine with varying concentrations of **C5:** (a) buffer blank; (b) guanine alone; (c) guanine and 10 µM **C5;** (d) guanine and 40 µM **C5;** and (e) guanine and 100 µM **C5**. (**A′**) Square-wave voltammetry of 0.5 mM guanine with varying concentrations of **C5** as in (A). (**B**) Cyclic voltammetry of 0.5 mM guanine in control experiments with varying volumes of ethanol: (a) buffer blank; (b) 0 µL; (c) 15 µL; (d) 60 µL; and (e) 150 µL. (**B′**) Square-wave voltammetry of 0.5 mM guanine in control experiments with varying volumes of ethanol: (a) buffer blank; (b) 0 µL; (c) 15 µL; (d) 60 µL; and (e) 150 µL. (**C**) Cyclic voltammetry of **C5** alone at: (a) 0 µM; (b) 10 µM; (c) 40 µM; and (d) 100 µM. (**C′**) Square-wave voltammetry of **C5** alone at: (a) 0 µM; (b) 10 µM; (c) 40 µM; and (d) 100 µM.

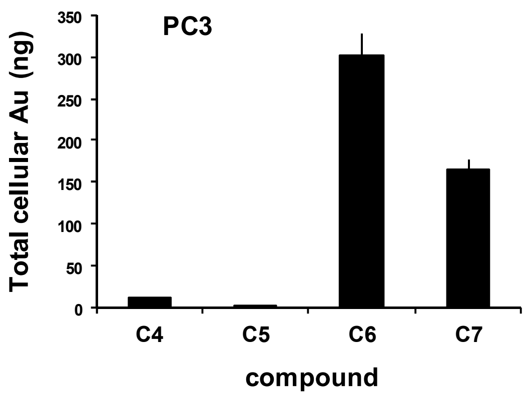

Figure S11. Uptake of selected gold(III) compounds by PC3 cells. PC3 cells (1 × 10^6 cells/dish) were treated for 2 h with 3 µM **C4, C5, C6** or **C7**, and internalized gold was determined by ICP mass spectrometry. Results are means and SD of three independent experiments.

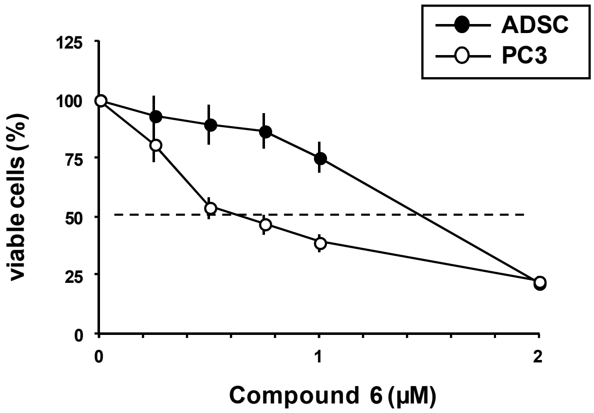

Figure S12. Growth inhibition curves for C6 in PC3 prostate cancer cells and adipose-derived stromal cells (ADSCs). Cell viability was determined with the MTT assay after 72 h drug treatment. Results are means and SD for three replicate wells from three independent experiments.

Table S1. Voltammetry peak potential (mV) of 100 µM compounds **(C1–C4**) with 1–10 µM lysozyme.

Compound	Technique	Lysozyme			
		0 µM	1 µM	4 µM	10 µM
**C1**	CV	0.73898	0.81421	0.83191	0.87616
**C1**	SWV	0.79338	0.80345	0.8387	0.86388
**C2**	CV	0.79208	0.80093	0.81863	0.83191
**C2**	SWV	0.78835	0.79338	0.80849	0.81352
**C3**	CV	0.79651	0.80978	0.82306	0.83633
**C3**	SWV	0.79338	0.80345	0.81352	0.82359
**C4**	CV	0.79651	0.80978	0.81863	0.82748
**C4**	SWV	0.78835	0.79338	0.80849	0.81352

CV, cyclic voltammetry; SWV, square-wave voltammetry.

Table S2. Voltammetry peak potential (mV) of 0.5 mM tryptophan with 10–100 µM compounds **(C5–C8).**

Compound	Technique	Compound			
		0 µM	10 µM	40 µM	100 µM
**C5**	CV	0.71243	0.72571	0.74341	0.74783
**C5**	SWV	0.70862	0.71365	0.73883	0.7489
**C6**	CV	0.71243	0.73013	0.73898	0.74341
**C6**	SWV	0.70862	0.71869	0.72372	0.72876
**C7**	CV	0.71243	0.72571	0.75668	0.78323
**C7**	SWV	0.70862	0.71365	0.72876	0.7338
**C8**	CV	0.71243	0.71686	0.72128	0.78323
**C8**	SWV	0.70862	0.70862	0.71365	0.71869

CV, cyclic voltammetry; SWV, square-wave voltammetry.

Table S3. Voltammetry peak potential (mV) of 0.5 mM guanine with 10–100 µM compounds **C1** and **C5.**

Compound	Technique	Compound			
		0 µM	10 µM	40 µM	100 µM
**C1**	CV	0.93811	0.96024	0.98236	1.0222
**C1**	SWV	0.93437	0.94444	0.95955	0.9948
**C5**	CV	0.93811	0.99564	1.0133	1.0753
**C5**	SWV	0.93437	0.9948	1.025	1.0502

CV, cyclic voltammetry; SWV, square-wave voltammetry.

© 2019 by the authors. Licensee MDPI, Basel, Switzerland. This article is an open access article distributed under the terms and conditions of the Creative Commons Attribution (CC BY) license (http://creativecommons.org/licenses/by/4.0/).
